# Bread Enriched With Legume Microgreens and Leaves—Ontogenetic and Baking-Driven Changes in the Profile of Secondary Plant Metabolites

**DOI:** 10.3389/fchem.2018.00322

**Published:** 2018-08-16

**Authors:** Rebecca Klopsch, Susanne Baldermann, Alexander Voss, Sascha Rohn, Monika Schreiner, Susanne Neugart

**Affiliations:** ^1^Leibniz Institute of Vegetable and Ornamental Crops, Grossbeeren, Germany; ^2^NutriAct–Competence Cluster Nutrition Research Berlin-Potsdam, Nuthetal, Germany; ^3^Institute of Nutritional Science, Department of Food Chemistry, University of Potsdam, Nuthetal, Germany; ^4^Institute for Food and Environmental Research (ILU) e. V., Nuthetal, Germany; ^5^Hamburg School of Food Science, Institute for Food Chemistry, Universität Hamburg, Hamburg, Germany

**Keywords:** ontogeny, microgreen, pea, lupin, flavonoid, carotenoid, thermal processing of food

## Abstract

Flavonoids, carotenoids, and chlorophylls were characterized in microgreens and leaves of pea (*Pisum sativum*) and lupin (*Lupinus angustifolius*) as these metabolites change during ontogeny. All metabolites were higher in the leaves for both species. Acylated quercetin and kaempferol sophorotrioses were predominant in pea. Genistein and malonylated chrysoeriol were predominant in lupin. Further, the impact of breadmaking on these metabolites using pea and lupin material of two ontogenetic stages as an added ingredient in wheat-based bread was assessed. In “pea microgreen bread” no decrease of quercetin was found with regard to the non-processed plant material. However kaempferol glycosides showed slight decreases induced by the breadmaking process in “pea microgreen bread” and “pea leaf bread.” In “lupin microgreen bread” no decrease of genistein compared to the non-processed plant material was found. Chrysoeriol glycosides showed slight decreases induced by the breadmaking process in “lupin microgreen bread” and “lupin leaf bread.” In all breads, carotenoids and chlorophylls were depleted however pheophytin formation was caused. Thus, pea and lupin microgreens and leaves are suitable, natural ingredients for enhancing health-promoting secondary plant metabolites in bread and may even be used to tailor bread for specific consumer health needs.

**Graphical Abstract:**
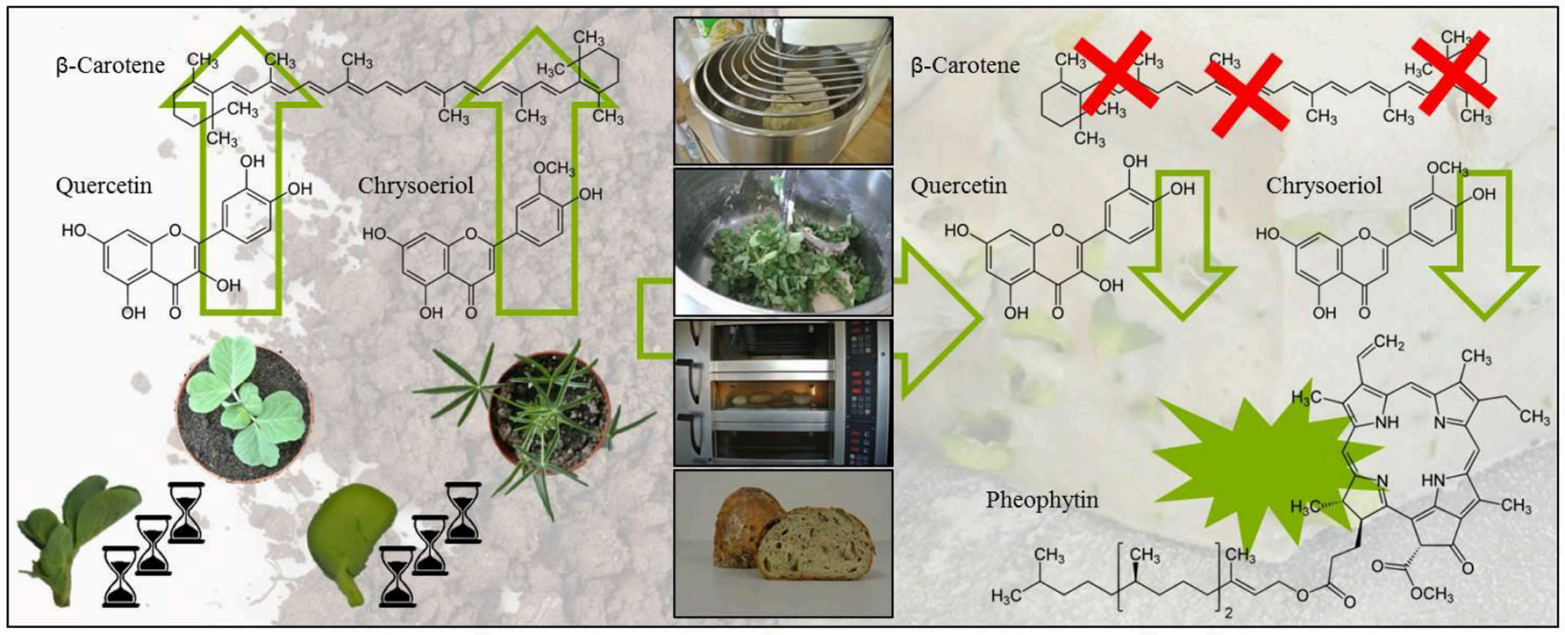
Impact of ontogeny and bread making on secondary plant metabolites in pea and lupin.

## Introduction

Fruits and vegetables contain health-promoting secondary plant metabolites that are not synthesized in mammalian organisms (Andersen and Markham, 2006). However, the overall fruit and vegetable consumption in industrialized nations with a Western food pattern is comparatively low, and strikingly, even below international recommendations of 250 g fruit and 375 g vegetables per day as recommended by the world health organization (World Health Organisation, 2018). Enhancing consumption of health-promoting secondary plant metabolites by means of fruit- and vegetable-enriched food products aimed at specifically increasing the current low intake of health-promoting substances would be both highly desirable. Bread is a frequently and highly consumed grain product in almost all types of Western diets. For example, up to four slices of bread per day are consumed in Northern European countries (Krems et al., 2013; Pot et al., 2015). Therefore, bread could be a suitable product to fortify with health-promoting secondary plant metabolites. To date, previous studies regarding bread-fortification were generally restricted to the addition of lyophilized plant powder or purified substances (Ranawana et al., 2016; Lin and Zhou, 2018). In this context, enrichment of wheat-based bread with freeze-dried quinoa leaf powder was reported to increase the antioxidant potential of the bread. However, detailed information on the specific substances that can increase antioxidant activity is still scarce (Ranawana et al., 2016). Lin and Zhou (2018) added pure quercetin to bread and found that the antioxidant and antiglycating potential of the bread increased, but that its rheological, and thus, baking properties were impaired. An alternative to adding such bread ingredients could be to use fresh, and unprocessed vegetative tissue such as microgreens (developed cotyledons) and leaves of the legume species pea (*Pisum sativum*) and lupin (*Lupinus angustifolius*). These species have the potential to be a new domestic source not only for vegetable protein, but also for antioxidant and thus health-promoting secondary plant metabolites such as flavonoids, carotenoids, and chlorophylls (Wang et al., 2008; Wojakowska et al., 2013; Santos et al., 2014; Bussler et al., 2015). Importantly, studies suggest that their consumption helps to prevent degenerative diseases and that these health-promoting properties are mostly attributed to their antioxidant properties conferred by the bioactivity of their secondary plant metabolites (Hsu et al., 2013; Frede et al., 2017; Kashyap et al., 2017; Romagnolo et al., 2017). The profile of flavonoids, carotenoids, and chlorophylls in leafy plant material are strongly affected by plant development stages that lead to immense variations in the concentration and composition of the secondary plant metabolites (Lefsrud et al., 2007; Aisyah et al., 2016). Finally, the breadmaking process affects the overall quality of this product, in terms of its characteristic texture, flavor, and color. The specific impact of thermal food processing depends on various factors concerning the plant material used, such as plant matrix, plant genotype, and also on the chemical structure of the specific secondary plant metabolite (Turkmen et al., 2006; Chaaban et al., 2017; Guillén et al., 2017). For example, modifications of flavonoids resulting from thermal food processing can be followed at hand their acylation and glycosylation patterns and the position of their sugar moieties (Rohn et al., 2007; Fiol et al., 2013; Chaaban et al., 2017). Moreover, carotenoids and chlorophylls can be affected due to thermal food processing (Turkmen et al., 2006; Guillén et al., 2017). Therefore, the first aim of the present study was to characterize the concentration and composition of flavonoids, carotenoids, and chlorophylls in particular, in peas and lupins in two different ontogenic stages (microgreens and leaves) as well as when being used in bread for enriching the concentration of secondary plant metabolites. With such new knowledge, we later on aim to fortify bread with vegetable plant material containing suitable secondary plant metabolites, with the ultimate goal of enhancing the bioactive and antioxidative properties of a highly consumed food product in a typical Western diet.

## Materials and methods

### Chemical and reagents

Ammonium acetate, genistein, kaempferol-3-*O*-glucoside, methanol, quercetin-3-*O*-glucoside, and *tert*-butyl methyl ether were purchased from Roth (Carl Roth GmbH, Karlsruhe, Germany). Acetic acid, 2-propanol, and dichloromethane were obtained from Merck (Merck KGaA, Darmstadt, Germany); acetonitrile from J. T. Baker (Fisher Scientific GmbH, Grießheim, Germany) and tetrahydrofurane (THF) from VWR (VWR International GmbH, Darmstadt, Germany). Standards of β-carotene, lutein, and zeaxanthin were from CaroNature (Carote*Nature* GmbH, Münsingen, Switzerland). Chlorophyll a and chlorophyll b were purchased from Sigma-Aldrich (Sigma Aldrich Chemie GmbH Taufkirchen, Germany). Chrysoeriol was obtained from Phytolab (Phytolab GmbH & Co. KG, Vestenbergsgreuth, Germany) and pheophytin a from LGC Standards (LGC Standards GmbH Wesel, Germany). All chemicals were of HPLC-MS-grade quality.

### Plant material

Two species from the plant family Fabaceae were grown for 7 and 14 days. For peas, the cultivar Blauwschokker Kapuzinererbse (Sperli GmbH, Everswinkel, Germany) and for lupins, the cultivar Dünge-Lupine (Kiepenkerl/Bruno Nebelung GmbH, Everswinkel, Germany) was chosen. Plants were grown in trays with soil (Einheitserde classic, Einheitserde Werkverband e.V., Germany) in a climate chamber. The pH of the soil was 5.9, N was 183 mg L^−1^, P_2_O_5_ was 135 mg L^−1^, K_2_O was 212 mg L^−1^, and salinity was 1.23 g L^−1^. The temperature setting was 22/18°C (day/night), with a light intensity of 500 μmol m^−2^ s^−1^ and a 12/12 h photoperiod, and air humidity of 70%. Plants were watered *ad libitum* and no fertilizer was added. After 7 days, microgreens (developed cotyledons) were harvested, while another batch of the same plants was grown further. Peas and lupins were harvested as five biological replicates (five microgreens per replicate), immediately frozen in liquid nitrogen, and then lyophilized. Fresh plant material was used for the breadmaking experiments. Peas and lupins not used for secondary metabolite analyses or breadmaking experiments were transferred in pots with a diameter of 8 cm and grown for another 7 days in soil in the climate chamber. Leaves of young plants (developmental stage: 14-d old/five true leaves developed) were harvested again as five biological replicates (leaves of five plants per replicate), immediately frozen in liquid nitrogen, and then lyophilized. Again, fresh plant material was used for breadmaking experiments.

### Breadmaking experiments

Four different types of fortified bread were prepared: (I) bread with pea microgreens; (II) bread with pea leaves; (III) bread with lupin microgreens; and (IV) bread with lupin leaves. The basis, which was also used as control, was a plain mixed wheat bread consisting of 41% wheat flour (Type 550, Baeko Hansa eG, Germany, Hamburg), 30% water, 8.5% rye flour (Type, 1150, Baeko Hansa eG, Germany, Hamburg), 18% sour dough [rye flour + starter culture (*Reinzucht-Sauerteig*, Ernst Boecker GmbH & Co. KG, Germany, Minden)], 1.5% salt (European Salt Company GmbH & Co. KG, Germany, Hannover), and 1.2% yeast (Uniferm GmbH & Co. KG, Germany, Werne). The breads were prepared as follows: dry and liquid ingredients were added and kneaded with a spiral mixer for 6 min (4 min fast: 1,360 rpm, 2 min slow: 690 rpm). For the fortified breads, 60 g fresh plant material (microgreens intact; leaves roughly chopped, 2 × 2 cm) was then added to 500 g raw dough, such that these breads were fortified with 10.7% of fresh plant material. Plant material was incorporated slowly with a spiral mixer (1 min) to avoid destruction of the plant matrix. The dough was subjected to rising for 40–50 min in a fermentation chamber (40–45°C, 85% air humidity), and finally, baked for 40 min at 230°C. After cooling, the breads were frozen at −40°C, lyophilized, and ground. Analyses of plant-enriched breads were conducted from the whole loaf (crumb and crust).

### Flavonoid extraction

For the analysis of flavonoid glycosides, samples were extracted according to Neugart et al. (2015) with slight modifications. Lyophilized and ground samples (plant material: 10 mg, bread: 50 mg) were extracted with 60% aqueous methanol (600 μL) for 40 min in a thermomixer at 1,400 rpm and 20°C. Samples were centrifuged at 19,000 × g and 20°C for 10 min, and the supernatants were collected in a 1.5 ml Eppendorf empty tube. This extraction procedure was repeated twice, but with 300 μL 60% aqueous methanol and 20 min extraction time. The combined supernatants were evaporated to dryness in a rotary evaporator. The residues were suspended in 200 μL 10% aqueous methanol and transferred to Spin-X tubes with a 0.22 μm cellulose acetate membrane (Corning® Costar® Spin-X®, Sigma Aldrich Chemical Co., St. Louis, MI). Samples were centrifuged at 850 × g and 20°C for 5 min and transferred to HPLC vials.

### Analysis of flavonoids by HPLC-DAD-ESI-MS^N^

Flavonoid glycoside separation and identification was performed with an Agilent HPLC series 1100 (Agilent Technologies Sales & Services GmbH & Co.KG, Waldbronn, Germany) consisting of a degasser, binary pump, autosampler, column oven, and a photodiode array detector. As the mass spectrometer (MS), an ion trap (Bruker amazon SL, Bruker, Bremen, Germany) with an electrospray ionization (ESI) ion source (negative ionization mode) was used. The parameters for HPLC-DAD-ESI-MS^n^ measurements were optimized based on the method of Neugart et al. (2015). The standards chrysoeriol, genistein, kaempferol-3-*O*-glucoside, and quercetin-3-*O*-glucoside were used to obtain external calibration curves ranging from 0.1 to 10 mg 100 mL^−1^. The MS^n^ experiments were performed in auto mode up to MS^3^ in a scan range from *m/z* 200 to 2000 with a target mass of *m/z* 500. The results were expressed in mg g^−1^ of fresh weight (FW), as mean ± standard deviation (SD) of five biological repetitions from each ontogenetic stage of the non-processed plant material and two biological repetitions (each three technical replicates thereof) of the control bread or each plant-enriched bread. Concentrations of flavonoids in plant-enriched breads refer to the proportion of plant material used for the enrichement (10.7% plant material), thereby ensuring that the results of the plant-enriched breads are comparable with the results of the plant concentration.

### Carotenoid and chlorophyll extraction

For the analysis of carotenoids and chlorophyll metabolites, sample material was extracted according to Baldermann et al. (2013) with slight modifications. Lyophilized and ground samples (plant material: 10 mg, bread: 100 mg) were extracted with 500 μL methanol/tetrahydrofuran (1:1) for 5 min in an Eppendorf ThermoMixer® C at 1,400 rpm and 20°C. Samples were centrifuged at 1,900 × *g* and 20°C for 5 min, and the supernatants were collected in a 1.5 ml Eppendorf empty tube. Briefly, this extraction procedure was repeated until the sample material was decolorized. The combined supernatants were evaporated to dryness under a stream of liquid nitrogen. The dry residues of the plant samples were suspended in dichloromethane (50 μL) and 2-propanol (200 μL), bread samples were dissolved in 20 μL dichloromethane and 180 μL 2-propanol, and then filtered through PTFE-filter tubes (0.2 μm, amchro GmbH, Hattersheim, Germany) by centrifugation at 850 × *g* and 20°C for 2 min and transferred to HPLC vials. Samples were measured immediately after extraction.

### Analysis of carotenoids and chlorophylls by UHPLC-ToF-MS

Qualitative and quantitative analysis of carotenoids and chlorophyll metabolites was performed with an Agilent Technologies 1290 Infinity II UHPLC (Agilent Technologies Sales & Services GmbH & Co. KG, Waldbronn, Germany) according to Schröter et al. (2017) with slight modifications. Analytes were separated by a C30 column (YMC Co. Ltd., Kyoto, Japan, YMC C30, 100 × 2.1 mm, 3 μm) at 20°C. Mobile phases were methanol/*tert*-butyl methyl ether/water (81:15:4, v/v/v) for solvent A and methanol/*tert*-butyl methyl ether/water (6:90:4, v/v/v) for solvent B. Ammonium acetate (20 mM) was added to the mobile phases to increase the ionization. Separation was performed using a flow rate of 0.2 mL min^−1^. The gradient for A/B was as follows: 10 min, 100% A; 45 min 80% A; 55 min 0% A; 57 min, 100% A. The compounds were identified on an Agilent Technologies 6230 TOF LC/MS equipped with a multimode ion source in positive mode. The gas temperature was set to 300°C at a flow rate of 8 L min^−1^, the vaporizer was set to 200°C, and the nebulizer pressure was set to 35 psig. The voltage was set to 3,500 V and a fragmentor voltage of 175 V was applied at a corona current of 4 μA. Standards of β-carotene, chlorophyll a, chlorophyll b, lutein, pheophytin a, and zeaxanthin were used for external calibration ranging from 0.2 to 474 ng on the column. Violaxanthin was calculated as neoxanthin equivalent. The MS detection was performed in scan range from *m/z* 100 to 1,700. The results were expressed in μg g^−1^ FW, as mean ± SD of five biological repetitions from each ontogenetic stage of the non-processed plant material and two biological repetitions (each three technical replicates thereof) of the control bread and each bread enriched with plant material. Concentrations of carotenoids and chlorophyll in plant-enriched breads refer to the proportion of plant material (10.7%), thereby ensuring that the results of the enriched breads are comparable with the results of the corresponding plants.

### Data handling and statistical analysis

Data were expressed as mean ± SD. The statistical analyses were carried out using Statistica 12 for Windows (Version 9.0, Statsoft Inc., Tulsa, OK). The differences between the two ontogenetic stages (microgreens and leaves) and samples used for breadmaking (non-processed plant material and plant-enriched bread) were tested by the student *t*-Test. Normal distribution of data in the different samples was tested (*t*-Test). Statistical significance was defined for *p* ≤ 0.05 (95% confidence level).

## Results and discussion

### Identification and quantification of flavonoids in pea microgreens and leaves

**Figure 1:**
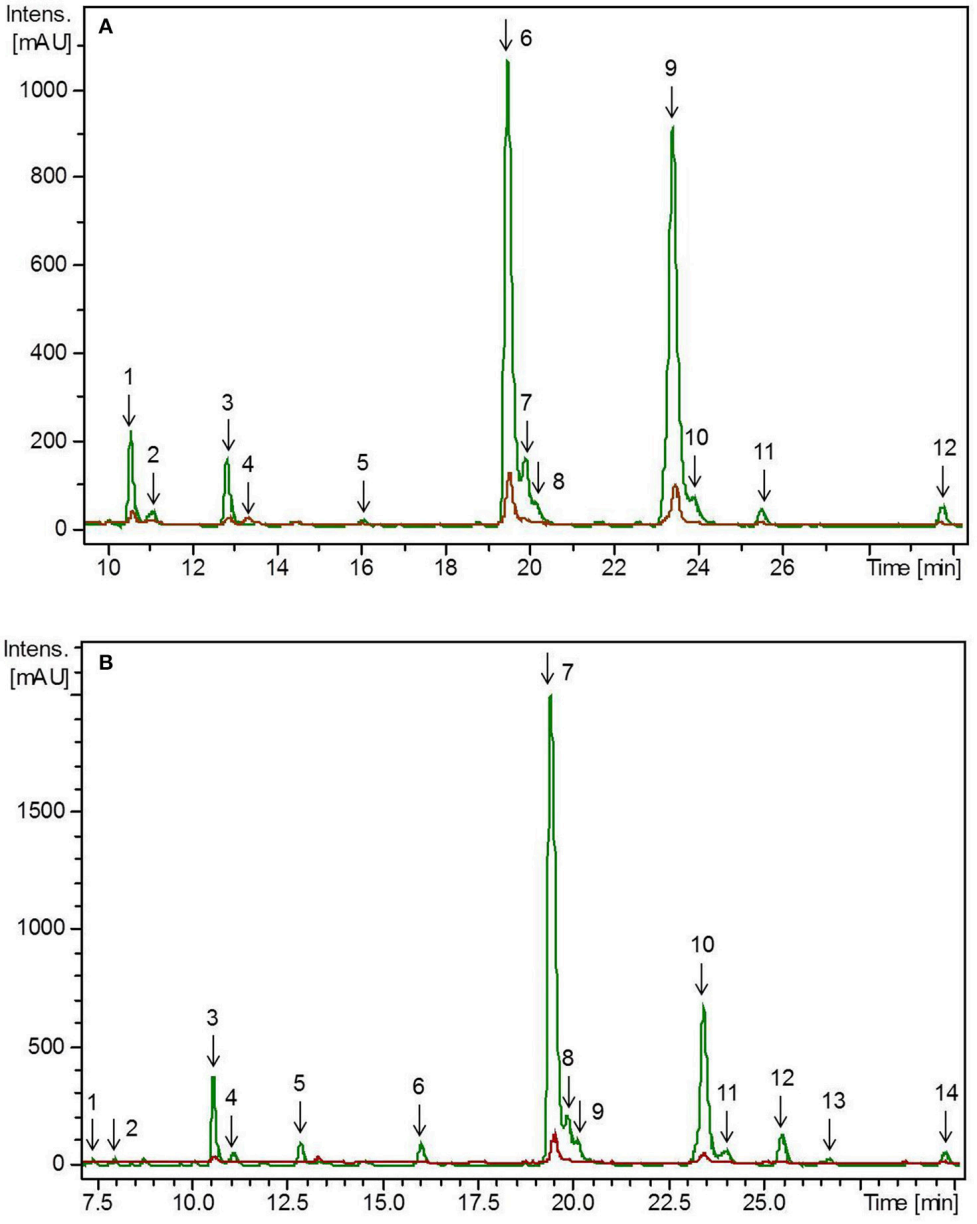
HPLC-chromatogram of flavonoid glycosides in **(A)** pea microgreens (green) vs. pea microgreen bread (brown) and **(B)** pea leaves (green) vs. pea leaf bread (brown). Intensity in mAU, extract at 330 nm. The bread contains 10.7% plant material, hence a dilution factor of 10.7% has to be taken into account. **(A)** Compounds are numbered in chronological order 1: Quercetin-3-*O*-sophorotriose 1, 2: Quercetin-3-*O*-sophorotriose 2, 3: Kaempferol-3*-O*-sophorotriose, 4: Kaempferol-3-*O*-sophoroside, 4: Quercetin-3-*O*-caffeoyl-sophorotriose, 5: Quercetin-3-*O*-coumaroyl-sophorotriose 1, 6: Quercetin-3-*O*-coumaroyl-sophorotriose 2, 7: Quercetin-3-*O*-feruloyl-sophorotriose, 8: Kaempferol-3-*O*-coumaroyl-sophorotriose 1, 9: Kaempferol-3-*O*-feruloyl-sophorotriose, 10: Quercetin-3*-O*-coumaroyl-sophorotriose 3, 11: Kaempferol-3-*O*-coumaroyl-sophorotriose 2 **(B)** Compounds are numbered in chronological order 1: Quercetin-3-*O*-sophorotriose 1, 2: Quercetin-3-*O*-sophorotriose 2, 3: Kaempferol-3-*O*-sophorotriose, 4: Quercetin-3-*O*-sophorotriose 2, 5: Quercetin-3-*O*-caffeoyl-sophorotriose, 6: Quercetin-3-*O*-coumaroyl-sophorotriose 1, 7: Quercetin-3-*O*-coumaroyl-sophorotriose 2, 8: Quercetin-3-*O*-feruloyl-sophorotriose, 9: Kaempferol-3-*O*-coumaroyl-sophorotriose 1, 9: Kaempferol-3-*O*-feruloyl-sophorotriose, 10: Quercetin-3-*O*-coumaroyl-sophorotriose 3, 11: Kaempferol-3-*O*-coumaroyl-sophorotriose.

All experiments (plant growth, breadmaking) were carried out in two plant growth trials. The initial values of flavonoids in the plants differed between these two independent experiments due to the natural biological variation depending mainly on ecophysiological factors such as temperature, light, soil composition. Thus, to achieve improved clarity, values of the first and second experiment were evaluated separately. To get an impression about the composition of the phenolic compounds in the plant materials, flavonoids were tentative identified by means of retention time and mass spectra obtained by HPLC-DAD-ESI-MS^n^ (Figure 1). In the first experiment, the total flavonoid concentration in pea microgreens was 3.31 mg g^−1^ FW, whereas the concentration in pea leaves was 4.37 mg g^−1^ FW (Table 1A). In the second, experiment the total flavonoid concentration in pea microgreens was 1.38 mg g^−1^ FW, whereas the concentration in pea leaves was 3.84 mg g^−1^ FW (Table 1B). In accordance with earlier studies, in both ontogenetic stages mainly flavonoids based on the flavonol aglycones kaempferol and quercetin were found (Bussler et al., 2015; Neugart et al., 2015). These flavonoids were tentatively identified by their deprotonated pseudomolecular ions (quercetin: *m/z* 301; kaempferol: *m/z* 285) (Supplemental Table S1) (Bussler et al., 2015; Neugart et al., 2015). With one exception, all quercetin and kaempferol glycosides identified were glycosylated with sophorotrioses, observable by the loss of 486 Da in MS^3^ (Table 1, Supplemental Table S1; Neugart et al., 2015). In general, aglycones have a higher antioxidant capacity compared to their corresponding glycosides. However, flavonoids are naturally glycosylated in plants (Andersen and Markham, 2006). Seven flavonoid glycosides were further acylated either with coumaric, caffeic, or ferulic acid, in the present study (Table 1). Transport of flavonoids between the membrane compartments, and thus, accumulation, as needed during plant growth, requires multiple modifications, e.g., glycosylation. Glycosylation of flavonoids increases their solubility, and thereby, facilitates their transport *in planta*. In addition, glycosylation can reduce flavonoid toxicity, and thus, protect membranes (Zhao and Dixon, 2010). In the first experiment, the total quercetin concentration was 2-fold higher in pea microgreens and 6-fold higher in pea leaves compared to the total kaempferol concentration (Table 1A). In the second experiment, the total quercetin concentration was 3-fold higher in pea microgreens and 5-fold higher in pea leaves (Table 1B). These increases are in accordance to earlier findings (Bussler et al., 2015; Neugart et al., 2015). As already described very often in the literature, quercetin and its derivatives have a higher radical scavenging and antioxidant capacity compared to kaempferol (and its derivatives) due to the catechol structure of the B-ring (Andersen and Markham, 2006; Fiol et al., 2013). Thus, quercetin could provide a higher level of protection against oxidative stress in plants and humans compared to kaempferol. In the present study, the concentration of non-acylated kaempferol derivatives decreased during plant development from microgreens to leaves. Considering the increase of quercetin from pea microgreens to leaves, as well as taking into account that kaempferol is the precursor of quercetin, a transformation of the kaempferol glycosides to quercetin glycosides can be assumed (Andersen and Markham, 2006; Saito et al., 2013). Acylation patterns of kaempferol and quercetin glycosides were inconsistent between pea microgreens and leaves. Acylation is known to promote a high diversity of flavonoids and this diversity is influenced by origin, quantity, and linkage, as well as type of the particular acyl groups (Andersen and Markham, 2006). Such a high diversity in the chemical structure confers different protective properties and implies a specific need for these functions such as varying requirements for different developmental stages (Andersen and Markham, 2006). In the first experiment, 11 out of 13 flavonoids identified were present in both developmental stages (Table 1A); in the second experiment, 10 out of 13 flavonoids were present (Table 1B). Earlier studies described hydroxycinnamic acid derivatives in 16-day-old pea seedlings and 8-week-old pea leaves (Bussler et al., 2015; Neugart et al., 2015). In contrast, hydroxycinnamic acid derivatives only occurred in trace amounts, in the present study (data not shown). Although the concentrations of quercetin and kaempferol aglycones and the quercetin/kaempferol ratio were in accordance to previous studies, the degree of hydroxylation and acylation varied between the different investigations (Bussler et al., 2015; Neugart et al., 2015). However, other studies have shown that polyphenols in general and flavonoid profiles in particular varied among different pea tissues, organs, and cultivars (Neugart et al., 2015; Šibul et al., 2016). The present study showed that quercetin glycosides were predominant in the investigated pea tissues, and that pea leaves accumulated a higher total flavonoid concentration compared to the microgreens.

**Table 1:** Average flavonoid concentration in pea microgreens (7 d) and pea leaves (14 d): Non-processed and in the pea-enriched bread.

		**Pea microgreens**			**Pea leaves**		
		**mg g**^**−1**^ **fresh weight**					
**(A) FIRST EXPERIMENT**							
Kaempferol-3-*O*-sophoroside	Non-processed	0.0054^A^	±	*0.0004*		ND
	After baking	0.0493^B^	±	*0.0025*	
Kaempferol-3-*O*-sophorotriose	Non-processed	0.1004^A, a^	±	*0.0086*	0.0070^b^	±	*0.0015*
	After baking	0.0636^B^	±	*0.0017*		ND
Kaempferol-3-*O*-coumaroyl-sophorotriose 1	Non-processed	0.9256^A, a^	±	*0.0937*	0.5394^A, b^	±	*0.0363*
	After baking	0.7558^B^	±	*0.0055*	0.2500^B^	±	*0.0340*
Kaempferol-3-*O*-coumaroyl-sophorotriose 2	Non-processed	0.0351^A, a^	±	*0.0034*	0.0332^a^	±	*0.0015*
	After baking	0.0309^B^	±	*0.0007*		ND
Kaempferol-3-*O*-feruloyl-sophorotriose	Non-processed	0.0427^A, a^	±	*0.0076*	0.0478^a^	±	*0.0033*
	After baking	0.0410^A^	±	*0.0006*		ND
Quercetin-3-*O*-sophorotriose 1	Non-processed	0.2175^A, a^	±	*0.0332*	0.3381^A, b^	±	*0.0275*
	After baking	0.3614^B^	±	*0.0045*	0.2336^B^	±	*0.0346*
Quercetin-3-*O*-sophorotriose 2	Non-processed	0.0494^A, a^	±	*0.0146*	0.0559^A, a^	±	*0.0121*
	After baking	0.1732^B^	±	*0.0045*	0.1169^B^	±	*0.0159*
Quercetin-3-*O*-sophorotriose 3	Non-processed		ND		0.0884^A^	±	*0.0133*
	After baking				0.0920^A^	±	*0.0107*
Quercetin-3-*O*-coumaroyl-sophorotriose 1	Non-processed	1.6031^A, a^	±	*0.2366*	2.7343^A, b^	±	*0.1157*
	After baking	1.2786^B^	±	*0.0282*	1.1946^B^	±	*0.1458*
Quercetin-3-*O*-coumaroyl-sophorotriose 2	Non-processed	0.2096^A, a^	±	*0.0274*	0.2467^b^	±	*0.0167*
	After baking	0.2657^B^	±	*0.0128*		ND
Quercetin-3-*O*-coumaroyl-sophorotriose 3	Non-processed	0.0511^A, a^	±	*0.0059*	0.1519^A, b^	±	*0.0088*
	After baking	0.1124^B^	±	*0.0018*	0.0784^B^	±	*0.0041*
Quercetin-3-*O*-caffeoyl-sophorotriose	Non-processed	0.0180^A, a^	±	*0.0024*	0.0684^A, b^	±	*0.0117*
	After baking	0.0688^B^	±	*0.0008*	0.0617^A^	±	*0.0041*
Quercetin-3-*O*-feruloyl-sophorotriose	Non-processed	0.0504^a^	±	*0.0086*	0.0757^b^	±	*0.0073*
	After baking		ND			ND
**(B) SECOND EXPERIMENT**							
Kaempferol-3-*O*-sophoroside	Non-processed	0.0027^A^	±	*0.0002*		ND
	After baking	0.0371^B^	±	*0.0027*			
Kaempferol-3-*O*-sophorotriose	Non-processed	0.0225^A, a^	±	*0.0027*		ND
	After baking	0.0303^B^	±	*0.0032*	
Kaempferol-3-*O*-coumaroyl-sophorotriose 1	Non-processed	0.2571^A, a^	±	*0.0244*	0.6125^A, b^	±	*0.0504*
	After baking	0.2231^A^	±	*0.0316*	0.2953^B^	±	*0.0158*
Kaempferol-3-*O*-coumaroyl-sophorotriose 2	Non-processed	0.0150^a^	±	*0.0018*	0.0444^b^	±	*0.0061*
	After baking		ND			ND
Kaempferol-3-*O*-feruloyl-sophorotriose	Non-processed	0.0099^a^	±	*0.0003*		ND
	After baking		ND	
Quercetin-3-*O*-sophorotriose 1	Non-processed	0.1052^A, a^	±	*0.0165*	0.2821^A, b^	±	*0.0394*
	After baking	0.1308^B^	±	*0.0078*	0.3072^A^	±	*0.0186*
Quercetin-3-*O*-sophorotriose 2	Non-processed	0.0297^A, a^	±	*0.0043*	0.0479^A, b^	±	*0.0090*
	After baking	0.0724^B^	±	*0.0035*	0.0540^A^	±	*0.0048*
Quercetin-3-*O*-sophorotriose 3	Non-processed		ND		0.1164^A^	±	*0.0366*
	After baking				0.1293^A^	±	*0.0080*
Quercetin-3-*O*-coumaroyl-sophorotriose 1	Non-processed	0.7769^A, a^	±	*0.1004*	2.4787^A, b^	±	*0.4208*
	After baking	0.6132^A^	±	*0.0669*	1.4110^B^	±	*0.0766*
Quercetin-3-*O*-coumaroyl-sophorotriose 2	Non-processed	0.0849^A, a^	±	*0.0048*	0.0938^b^	±	*0.0078*
	After baking	0.0920^A^	±	*0.0077*		ND
Quercetin-3-*O*-coumaroyl-sophorotriose 3	Non-processed	0.0375^A, a^	±	*0.0049*	0.1265^A, b^	±	*0.0200*
	After baking	0.0548^B^	±	*0.0017*	0.0545^B^	±	*0.0053*
Quercetin-3-*O*-caffeoyl-sophorotriose	Non-processed	0.0145^a^	±	*0.0023*	0.0185^A, b^	±	*0.0037*
	After baking		ND		0.0359^B^	±	*0.0025*
Quercetin-3-*O*-feruloyl-sophorotriose	Non-processed	0.0209^a^	±	*0.0017*	0.0165^b^	±	*0.0021*
	After baking		ND			ND

*Results are presented as means ± SD (Italic values, n = 5) in mg g^−1^ fresh weight. ND, Not detected. Students t-test by Statistica at a significance level of p ≤ 0.05. Capital letters label significant differences between the non-processed plant material and plant material after the baking process within in the same development stage. Small letters label significant differences between non-processed microgreens and leaves*.

### Identification and quantification of flavonoids in pea-enriched bread

**Figure 2:**
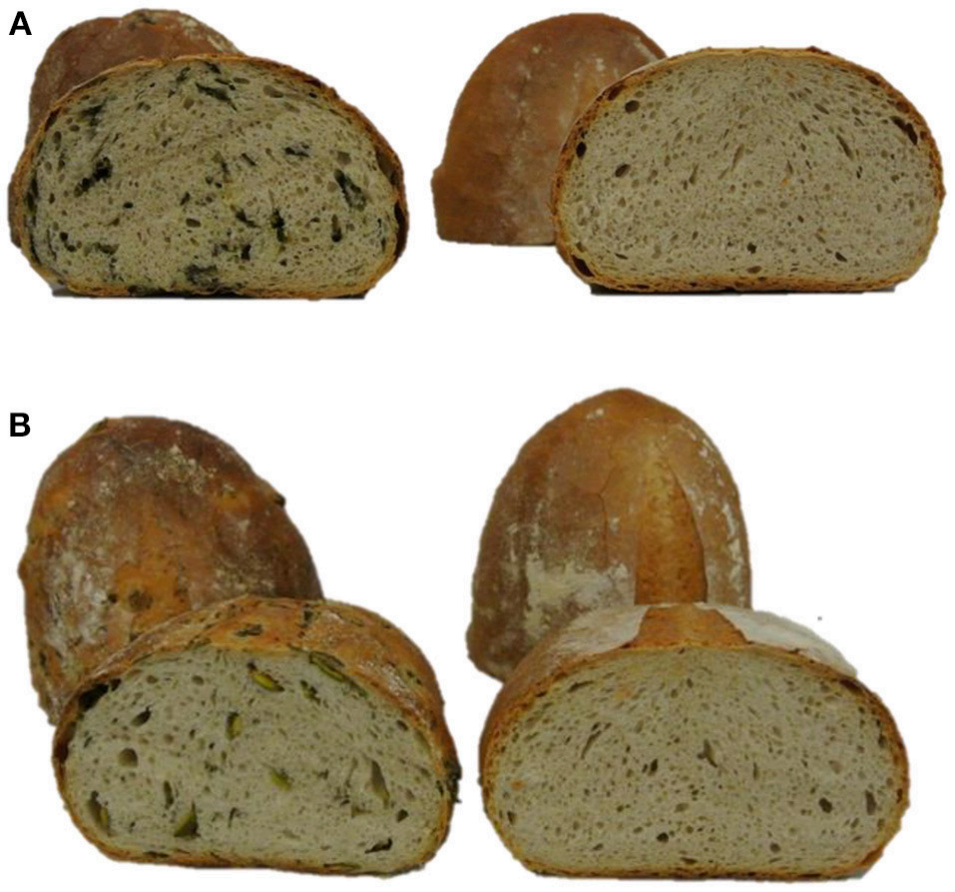
Breads fortified with plant material and reference bread. **(A)** Pea microgreen bread (left) and reference bread (right); **(B)** Lupin microgreen bread (left) and reference bread (right).

Hedonic tastings (5–7 persons) revealed only minor to no differences in the taste of the breads enriched with plant material compared to the control bread. Taste differences, if any, were described as, “green” or “grassy.” The plant material slightly improved chewing ability, freshness, and juiciness of the bread crumb. No effect on dough color was determined. However, the color of the plant material changed from a fresh green in the raw dough to an olive green in the baked bread (Figure 2). This was probably caused by chlorophyll depletion (Turkmen et al., 2006). Microgreens of pea and lupin reduced the bread volume by only 7–15% compared to the control bread. Microgreens showed no effect on the texture of the bread crumb and porosity. However, leaves slightly disrupted the texture of the crumb and the pores of the bread (data not shown). To follow the fate of the flavonoid profile during a food process, non-processed pea material from the day of breadmaking, pea-enriched breads, and control breads were analyzed for their flavonoid profile. None of the flavonoids present in the plant material were found in the control bread. In the bread with the pea material, only the one with the pea leaf material had a significant decrease in the total flavonoid concentration (Table 1, Figure 1). When using the plant material from the first experiment, 97% of the total flavonoid concentration quantified in non-processed pea microgreens, were found in the “pea microgreen bread.” In the “pea leaf bread” of the first experiment, only 46% of the initial total flavonoid concentration of the non-processed pea leaves was found (Table 1A). In the second experiment, 91% of the total flavonoid concentration quantified in non-processed pea microgreens, were found in the “pea microgreen bread.” In the “pea leaf bread” from the second experiment, 60% of the initial total flavonoid concentration was found (Table 1B). The structural peculiarities of the flavonoids were equally influenced by breadmaking in both experiments. Also the dimension of losses was the same in both experiments. Even though there was a significant decrease in total kaempferol and quercetin concentration, the quercetin/kaempferol ratio increased in “pea leaf bread” compared to non-processed pea leaves. This implies a lower loss of quercetin glycosides when thermally processed. As shown by Ewald et al. (1999), different thermal processing treatments, including blanching, boiling, microwaving, and roasting, of onions also led to a slight decrease of quercetin compared to kaempferol concentrations. When comparing a wide variety of different heat treatments (blanching, boiling, microwaving, frying, steaming), boiling always induced the highest decrease, thereby implying losses that are also due to leaching effects (Ewald et al., 1999; Baardseth et al., 2010). In the present study, some results were inconsistent between pea microgreens and leaves. This discrepancy was possibly due to differences in the pea matrix rigidity since losses due to leaching were excluded since the pea material was incorporated into dough. In the present study, a significant decrease of all acylated kaempferol glycosides in pea-enriched breads compared to non-processed pea microgreens and leaves was detected (Table 1, Figure 1). Moreover, the only sophoroside identified in pea microgreens, namely kaempferol-3-*O*-sophoroside, increased significantly in “pea microgreen bread” compared to the non-processed microgreens in both experiments. Fiol et al. (2013) also found a decrease of highly glycosylated flavonoid glycosides in favor of an increase or formation of structurally related compounds with one or two fewer sugar moieties after cooking kale (*Brassica oleracea* var. *sabellica*). These increases could be probably due to deglycosylation and deacylation of kaempferol glycosides in pea microgreens (Rohn et al., 2007; Fiol et al., 2013). Previous work from Buchner et al. (2006) and Rohn et al. (2007) also showed that the stability of quercetin glycosides is dependent on their glycoside moiety as well as their position, but also highly depending on the thermal treatment used (aqueous vs. dry treatment). In accordance with the results of the present study, this suggests that the thermostability of flavonoids is dependent on their glycosylation and acylation status. For non-acylated quercetin compounds, a general increase in pea-enriched breads compared to non-processed pea materials was observed (Table 1). Also Chaaban et al. (2017) proposed that glycosylated flavonoids have a higher stability during thermal treatment. Further, the thermal response of quercetin glycosides was shown to vary depending on the glycosylation status, and even, specific isomers, as it was also the case in the present study for all three isomers of quercetin-3-*O*-coumaroyl-sophorotriose (Table 1, Figure 1). Juániz et al. (2016) suggested that the general increase of polyphenols during different heat treatments of yellow onion (*Allium cepa*), Italian sweet green pepper (*Capsicum annuum*), and cardoon stalks (*Cynara cardunculus* L.) was driven by thermally-induced destruction of cell walls and subcellular compartments. Thus, in relation to the present study, inconsistent results could be due to a heterogeneous distribution of the plant material in the dough/bread, and thereby, a heterogeneous thermally-induced disruption of cell structures. This hypothesis is consistent with the fact that during breadmaking, the plant material is exposed to both, mechanical (e.g., kneading) and thermal (e.g., baking) stress. Depending on the distribution of the plant material in the dough, these processes may have fractured the cell structures to a varying extent. Other studies have focused on differently processed plant material, e.g., fresh, lyophilized, and ground, or isolated flavonoids which could explain differing results (Rohn et al., 2007; Fiol et al., 2013; Chaaban et al., 2017). Extensive research on the health-promoting properties of kaempferol and quercetin has been carried out over the years, and both have been shown to have the potential to reduce the risk of suffering from a variety of diseases and disorders, from anticarcinogenic, anti-inflammatory, anti-obesity, and antiviral effects (Wang et al., 2016; Kashyap et al., 2017). Finally, taking the higher initial flavonoid concentration of pea leaves into account, one slice of bread (50 g) enriched with either pea microgreens or leaves contained on average 6–15 mg of flavonoids.

### Identification of flavonoids in lupin microgreens and leaves

**Figure 3:**
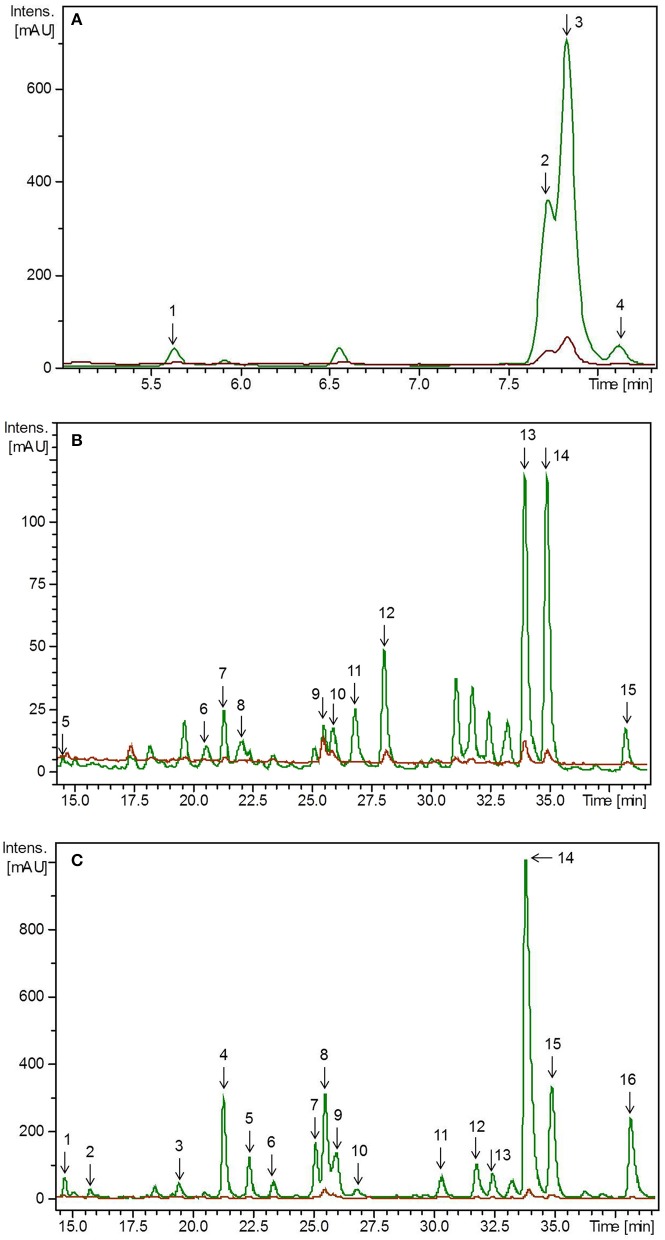
HPLC-chromatogram of flavonoid glycosides in **(A)** lupin microgreens (green) vs. lupin microgreen bread (brown) in the time range 4–8 min, **(B)** lupin microgreens (green) vs. lupin microgreen bread (brown) in the time range 15–40 min and **(C)** lupin leaves (green) vs. lupin leaf bread (brown). Intensity in mAU, extract at 330 nm. The bread contains 10.7% plant material, hence a dilution factor of 10.7% has to be taken into account. **(A)** Compounds are numbered in chronological order 1: 2'-Hydroxygenistein-*C*-diglucoside malonylated, 2: Genistein-*C*-diglucoside–xyloside 1, 3: Genistein-*C*-diglucoside–xyloside 2, 4: Genistein-*C*-diglucoside–xyloside 3 **(B)** Compounds are numbered in chronological order 5: Chrysoeriol glucoside–xylosyl glucoside 1, 6: Chrysoeriol glucoside–xylosyl glucoside 2, 7: Chrysoeriol glucoside–xylosyl glucoside malonylated 1, 8: Chrysoeriol glucoside–xylosyl glucoside malonylated 2, 9: Chrysoeriol glucoside–xylosyl glucoside dimalonylated 1, 10: Chrysoeriol glucoside–xylosyl glucoside dimalonylated 2, 11: Chrysoeriol glucoside–xylosyl glucoside dimalonylated 3, 12: Chrysoeriol glucoside–xylosyl glucoside dimalonylated 4, 13: Chrysoeriol-xylosyl glucoside malonylated, 14: Chrysoeriol-*O*-glucoside malonylated, 15: Chrysoeriol-xylosyl glucoside dimalonylated. **(C)** Compound are numbered in chronological order 1: Chrysoeriol glucoside-xylosyl glucoside 1, 2: Chrysoeriol glucoside-xylosyl glucoside 2, 3: Chrysoeriol glucoside-xylosyl glucoside malonylated 1, 4: Chrysoeriol glucoside-xylosyl glucoside malonylated 2, 5: Chrysoeriol glucoside-xylosyl glucoside malonylated 3, 6: Chrysoeriol glucoside-xylosyl glucoside malonylated 4, 7: Chrysoeriol glucoside-xylosyl glucoside dimalonylated 1, 8: Chrysoeriol-*O*-xylosyl glucoside, 9: Chrysoeriol glucoside-xylosyl glucoside dimalonylated I2, 10: 2′-Hydroxygenistein-*C*-glucosid malonylated, 11: Chrysoeriol-xylosyl glucoside malonylated 1, 12: Chrysoeriol-xylosyl glucoside malonylated 2, 13: Genistein-*O*-xylosyl glucoside malonylated, 14: Chrysoeriol-xylosyl glucoside malonylated 3, 15: Chrysoeriol-*O*-glucoside malonylated, 16: Chrysoeriol-xylosyl glucoside dimalonylated.

Similarly to the pea materials, all experiments were carried out twice. Accordingly, the initial values of flavonoids in the plants differed between these two independent experiments due to biological variations. Thus, to achieve improved clarity, values of the first and second experiment, have been evaluated separately. Flavonoids were tentatively identified by means of retention time and mass spectra obtained by HPLC-DAD-ESI-MS^n^ as well as by comparing the obtained mass spectra with the literature (Figure 3, Supplemental Table S2) (Kachlicki et al., 2008; Duenas et al., 2009; Wojakowska et al., 2013). In the first experiment, the average flavonoid concentration in lupin microgreens was 2.08 mg g^−1^ FW and increased in lupin leaves to 4.55 mg g^−1^ FW (Table 2A). In the second experiment the average flavonoid concentration in lupin microgreens was 0.88 mg g^−1^ FW and increased in lupin leaves to 3.41 mg g^−1^ FW (Table 2B). Three different flavonoid aglycones were tentatively identified by their deprotonated pseudomolecular ions. Two of them were assigned to the group of isoflavones (genistein *m/z* 269 and 2'-hydroxygenistein *m/z* 187) and one to the group of flavones (chrysoeriol *m/z* 299) (Supplemental Table S2) (Duenas et al., 2009; Wojakowska et al., 2013). Aglycones were glycosylated with different sugar moieties such as glucose, xylose, or combinations thereof. As described in previous studies, genistein, 2′-hydroxygenistein, and chrysoeriol glycosides were, if present, acylated with one or two molecules of malonic acid (Table 2) (Kachlicki et al., 2008; Wojakowska et al., 2013). Malonic acid was also found as the prevailing substituent in 3- to 14-week-old lupin leaves and other organs (roots, stems, and inflorescences), whereby esterification with malonic acid was also widespread in lupin (García-López et al., 2006; Wojakowska et al., 2013). These observations indicate the occurrence of malonylation as age-, organ-, and species-independent. Malonylation is assumed to maintain flavonoids in the vacuole, protect flavonoid glycosides from enzymatic degradation by glycosidases or oxidases, and support their intracellular transport (Zhao and Dixon, 2010). In the present study, a shift in the chrysoeriol/genistein ratio due to a roughly 8-fold higher concentration of chrysoeriol in lupin leaves compared to microgreens was found (Table 2). In line with this finding, increasing isoflavone (genistein and 2′-hydroxygenistein) concentrations during germination of lupin seeds were reported previously (Aisyah et al., 2016). Of note is that isoflavones in general and genistein and 2′-hydroxygenistein derivatives in particular can exhibit antimicrobial activity and that their synthesis during germination is possibly assigned to their effect as phytoalexin and phytoanticipin on the vulnerable seedling (Zhang et al., 2008). In the present study, a significant decrease of the genistein derivatives' concentrations from day 7 to 14 of lupin development was found. This observation is consistent with the findings of Katagiri et al. (2000), who also detected a decrease in genistein derivative concentrations from day 9 to 13 in this species. Moreover, higher chrysoeriol concentrations compared to genistein ones was found in 3-week-old lupin leaves (Kachlicki et al., 2008). Generally, chrysoeriol glycosides increased in lupin leaves compared to microgreens. Five different chrysoeriol glycosides were detected exclusively in lupin leaves, suggesting their necessity in the later stages of plant development (Table 2). Chrysoeriol has antioxidant activitiy and possesses a high antioxidant potential, which is conferred by the presence and position of the phenolic hydroxyl group (Mishra et al., 2003; Nascimento et al., 2014). As already described above, glycosylation is also known to influence flavonoid's antioxidant activity (Mishra et al., 2003). Therefore, an increase of chrysoeriol glycosides during plant development might result from a severe need for antioxidant activity in developing plants compared to seedlings, as leaves have a higher surface area that is exposed to light. This hypothesis is substantiated by the observation that lupin leaves had a higher concentration and diversity of chrysoeriol glycosides compared to microgreens (Table 2). Nevertheless, in lupin, the occurrence of genistein substituents and malonic acid was generally consistent. Taking the present as well as previous studies into account, flavonoid composition and concentration appears to be influenced by plant developmental stage, organ, as well as species and cultivar (García-López et al., 2006; Wojakowska et al., 2013). Finally, even though the isoflavone (genistein) concentration was higher in lupin microgreens, the overall total flavonoid concentration was higher in lupin leaves.

**Table 2:** Average flavonoid concentration in lupin microgreens (7 d) and lupin leaves (14 d): Non-processed and in the lupin fortified bread.

		**Lupin microgreens**			**Lupin leaves**		
		**mg g**^**−1**^ **fresh weight**					
**(A) FIRST EXPERIMENT**							
Chrysoeriol*-O*-glucoside malonylated	Non-processed	0.1868^A, a^	±	*0.0151*	0.5204^A, b^	±	*0.0938*
	After baking	0.0608^B^	±	*0.0108*	0.0968^B^	±	*0.0066*
Chrysoeriol glucoside-xylosyl glucoside 1	Non-processed	0.0286^a^	±	*0.0023*	0.0134^A, b^	±	*0.0019*
	After baking		ND		0.0588^B^	±	*0.0006*
Chrysoeriol glucoside–xylosylglucoside 2	Non-processed	0.0032^a^	±	*0.0005*	0.0414^A, b^	±	*0.0022*
	After baking		ND		0.0368^B^	±	*0.0022*
Chrysoeriol glucoside–xylosyl glucoside malonylated 1	Non-processed		ND		0.0269	±	*0.0021*
	After baking					ND
Chrysoeriol glucoside–xylosyl glucoside malonylated 2	Non-processed		ND		0.0417	±	*0.0050*
	After baking		ND			ND
Chrysoeriol glucoside–xylosyl glucoside malonylated 3	Non-processed	0.0292^a^	±	0.0043	0.3618^A, b^	±	*0.0495*
	After baking		ND		0.0468^B^	±	*0.0011*
Chrysoeriol glucoside–xylosyl glucoside malonylated 4	Non-processed	0.0077^a^	±	0.0013	0.1149^A, b^	±	*0.0113*
	After baking		ND		0.0307^B^	±	*0.0009*
Chrysoeriol glucoside–xylosyl glucoside dimalonylated 1	Non-processed	0.0095^a^	±	*0.0008*	0.2052^A, b^	±	*0.0402*
	After baking		ND		0.0335^B^	±	*0.0009*
Chrysoeriol glucoside–xylosyl glucoside dimalonylated 2	Non-processed	0.0102^a^	±	*0.0012*	0.2094^A, b^	±	*0.0202*
	After baking		ND		0.2605^B^	±	*0.0052*
Chrysoeriol glucoside–xylosyl glucoside dimalonylated 3	Non-processed	0.0263^A, a^	±	*0.0031*		ND
	After baking	0.0686^B^	±	*0.0141*	
Chrysoeriol glucoside–xylosyl glucoside dimalonylated 4	Non-processed	0.0309^A, a^	±	*0.0030*	0.0501^B^	±	0.0034
	After baking	0.0545^B^	±	*0.0112*		ND
Chrysoeriol-*O*-xylosylglucoside	Non-processed		ND		0.3045^A^	±	*0.0562*
	After baking				0.0995^B^	±	*0.0021*
Chrysoeriol-xylosyl glucoside malonylated 1	Non-processed		ND		0.0540^A^	±	*0.0136*
	After baking				0.0540^A^	±	*0.0009*
Chrysoeriol-xylosyl glucoside malonylated 2	Non-processed		ND		0.1451	±	*0.0160*
	After baking					ND
Chrysoeriol-xylosyl glucoside malonylated 3	Non-processed	0.1816^A, a^	±	0.0246	1.7694^A, b^	±	*0.1804*
	After baking	0.0703^B^	±	0.0153	0.2323^B^	±	*0.0031*
Chrysoeriol-xylosyl glucoside dimalonylated	Non-processed	0.0251^a^	±	*0.0032*	0.3652^A, b^	±	*0.0435*
	After baking		ND		0.0313^B^	±	*0.0008*
Genistein-*C*-diglucoside–xyloside 1	Non-processed	0.4732^A^	±	*0.0295*		ND
	After baking	0.4571^A^	±	*0.0704*	
Genistein-*C*-diglucoside–xyloside 2	Non-processed	0.9904^A^	±	*0.0574*		ND
	After baking	1.0068^A^	±	*0.1595*	
Genistein-*C*-diglucoside–xyloside 3	Non-processed	0.0372^A^	±	*0.0034*		ND
	After baking	0.0912^B^	±	*0.0095*	
Genistein-*O*-xylosyl glucoside malonylated	Non-processed		ND		0.2593	±	*0.0406*
	After baking					ND
2'-Hydroxygenistein-*C*-glucoside malonylated	Non-processed		ND		0.0696	±	*0.0118*
	After baking					ND
2'-Hydroxygenistein-*C*-diglucoside malonylated	Non-processed	0.0377	±	*0.0073*		ND
	After baking		ND	
**(B) SECOND EXPERIMENT**							
Chrysoeriol*-O*-glucoside malonylated	Non-processed	0.1224^A, a^	±	*0.0194*	0.5017^A, b^	±	*0.0350*
	After baking	0.0451^B^	±	*0.0128*	0.1276^B^	±	*0.0066*
Chrysoeriol glucoside-xylosyl glucoside 1	Non-processed	0.0018^a^	±	0.0001	0.0164^A, b^	±	*0.0016*
	After baking		ND		0.0859^B^	±	*0.0052*
Chrysoeriol glucoside–xylosyl glucoside 2	Non-processed		ND		0.0399^A, b^	±	*0.0042*
	After baking				0.0485^B^	±	*0.0037*
Chrysoeriol glucoside–xylosyl glucoside malonylated 1	Non-processed		ND		0.0191	±	*0.0020*
	After baking					ND
Chrysoeriol glucoside–xylosyl glucoside malonylated 2	Non-processed		ND		0.0351	±	*0.0031*
	After baking					ND
Chrysoeriol glucoside–xylosyl glucoside malonylated 3	Non-processed	0.0345^a^	±	0.0116	0.2205^A, b^	±	*0.0298*
	After baking		ND		0.0583^B^	±	*0.0025*
Chrysoeriol glucoside–xylosyl glucoside malonylated 4	Non-processed	0.0098^a^	±	0.0029	0.0806^A, b^	±	*0.0086*
	After baking		ND		0.0342^B^	±	*0.0011*
Chrysoeriol glucoside–xylosyl glucoside dimalonylated 1	Non-processed	0.0064^a^	±	*0.0016*	0.0976^A, b^	±	*0.0152*
	After baking		ND		0.0433^B^	±	*0.0030*
Chrysoeriol glucoside–xylosyl glucoside dimalonylated 2	Non-processed	0.0162^a^	±	*0.0054*	0.1405^A, b^	±	*0.0129*
	After baking		ND		0.4853^B^	±	*0.0253*
Chrysoeriol glucoside–xylosyl glucoside dimalonylated 3	Non-processed	0.0181^A, a^	±	*0.0056*		ND
	After baking	0.0594^B^	±	*0.0190*	
Chrysoeriol glucoside–xylosyl glucoside dimalonylated 4	Non-processed	0.0205^A, a^	±	*0.0040*	0.0339^B^	±	0.0034
	After baking	0.0344^B^	±	*0.0067*		ND
Chrysoeriol-*O*-xylosyl glucoside	Non-processed		ND		0.2517^A^	±	*0.0243*
	After baking				0.1604^B^	±	*0.0227*
Chrysoeriol-xylosyl glucoside malonylated 1	Non-processed		ND		0.0553^A^	±	*0.0051*
	After baking				0.0781^B^	±	*0.0028*
Chrysoeriol-xylosyl glucoside malonylated 2	Non-processed		ND		0.1107	±	*0.0090*
	After baking					ND
Chrysoeriol-xylosyl glucoside malonylated 3	Non-processed	0.1799^A, a^	±	0.0511	1.2947^A, b^	±	*0.1041*
	After baking	0.0764^B^	±	0.0331	0.3836^B^	±	*0.0133*
Chrysoeriol-xylosyl glucoside dimalonylated	Non-processed	0.0357^a^	±	*0.0115*	0.2842^A, b^	±	*0.0225*
	After baking		ND		0.0370^B^	±	*0.0049*
Genistein-*C*-diglucoside–xyloside 1	Non-processed	0.1300^A^	±	*0.0295*		ND
	After baking	0.1289^A^	±	*0.0190*	
Genistein-*C*-diglucoside–xyloside 2	Non-processed	0.2795^A^	±	*0.0450*		ND
	After baking	0.2616^A^	±	*0.0441*	
Genistein-*C*-diglucoside–xyloside 3	Non-processed	0.0137^A^	±	*0.0016*		ND
	After baking	0.0285^B^	±	*0.0028*	
Genistein-*O*-xylosylglucoside malonylated	Non-processed		ND		0.1992	±	*0.0209*
	After baking					ND
2'-Hydroxygenistein-*C*-glucoside malonylated	Non-processed		ND		0.0258	±	*0.0295*
	After baking					ND
2'-Hydroxygenistein-*C*-diglucoside malonylated	Non-processed	0.0078	±	*0.0006*		ND
	After baking		ND	

*Results are presented as means ± SD (Italic values, n = 5) in mg g^−1^ fresh weight. ND, Not detected. Students t-test by Statistica at a significance level of p ≤ 0.05. Capital letters label significant differences between the non-processed plant material and plant material after the baking process within in the same development stage. Small letters label significant differences between non-processed microgreens and leaves*.

### Identification and quantification of flavonoids in lupin-enriched bread

Non-processed plant material from the day of breadmaking, lupin-enriched breads, and control breads were analyzed with regard to their flavonoid concentration. None of the flavonoids present in the plant material could be detected in the control bread. Total flavonoid concentration was lower in lupin-enriched breads compared to the non-processed plant material (Table 2, Figure 3). In the first experiment, the “lupin microgreen bread” contained 87% and the “lupin leaf bread” 22% of the total flavonoid concentration quantified in the corresponding non-processed lupin materials (Table 2A). In the second experiment, the “lupin microgreen bread” contained 72% and the “lupin leaf bread” 45% of the total flavonoid concentration quantified in the corresponding non-processed lupin materials (Table 2B). Bread production equally influenced the structural peculiarities of the flavonoids in both experiments. Also the dimension of losses was the same in both experiments. With regard to total chrysoeriol concentration, there was a decrease in baked lupin material compared to non-processed lupin material, whereas for the total genistein concentration of non-processed lupin microgreens and the “lupin microgreen bread,” no difference was found. Genistein glycosides were only identified in the non-processed lupin leaves, but not in the “lupin leaf bread.” Grün et al. (2001) reported that heat treatments had only little impact on the genistein concentration, whereas daidzein and its conjugates changed due to modifications of their specific glycosides. However, these isoflavonoids (daidzein, glycitin) were shown to be stable at temperatures around 100°C (Grün et al., 2001; Ungar et al., 2003). Purified β-glucoside genistein (genistein-7-*O*-glucoside) was shown to be stable up to 135°C (Xu et al., 2002). During the baking process in our study, the temperature in the bread crumbs did not increase higher than 98°C, whereas in the bread crust, it can reach temperatures between 160 and 180°C (Acker et al., 2013). Genistein derivatives were expected to be stable during baking, and indeed, total genistein concentration did not change after baking (Table 2). In previous studies, malonyl glycosides of genistein were shown to be unstable during heat treatment, resulting in decarboxylation and de-esterification as well as an increase in acetyl glycosides and β-glycosides (Ungar et al., 2003). In the present study, malonylated genistein and 2'-hydroxygenistein glycosides were not detectable in the lupin breads. In the “lupin leaf bread,” genistein derivatives were not even detectable at all (Table 2, Figure 3). The stability of chrysoeriol under heat treatment (100°C) was shown to be pH dependent in aqueous solutions with a high stability at pH 3 (Hostetler et al., 2013). In the present study, the pH of the dough was approximately 3.7 with a comparatively low concentration of free water. Therefore, a certain level of thermostability of chrysoeriol glycosides or occurrence of chrysoeriol aglycones in the baked bread could be expected. From 15 chrysoeriol glycosides identified in non-processed lupin microgreens, only four (two monoacylated and two diacylated with malonic acid) were also found in the “lupin microgreen bread” (Table 2, Figure 3). Moreover, the two monoacylated chrysoeriol glycosides showed a decrease and the two diacylated chrysoeriol glycosides an increase in the “lupin microgreen bread” compared to non-processed lupin microgreens (Table 2). Hence, acylation could have an impact on the thermostability of chrysoeriol. As already described for the pea material, the thermostability of flavonoid glycosides is known to be dependent on their structure, such as their glycosylation and acylation status, and glycosylated flavonoids might have a higher thermostability (Rohn et al., 2007; Fiol et al., 2013; Chaaban et al., 2017). However, the majority of the flavonoids detected in lupin microgreens and leaves showed a decrease after breadmaking (Table 2, Figure 3). Similar to the results obtained for the pea breads, discrepancies in detection levels might result from a heterogeneous distribution of lupin tissue in the dough/bread, and thereby, a heterogeneous fracturing of cell walls/compartments during breadmaking (Juániz et al., 2016). The “lupin microgreen bread” contained mainly isoflavone genistein glycosides, whereas the “lupin leaf bread” contained only flavone chrysoeriol glycosides. Isoflavonoids, such as genistein, can have estrogen-like effects, as they can bind to estrogen receptors due to their structural similarities with the human female hormone 17-β-estradiol. Thus, they have implications for, e.g., breast cancer (Xu et al., 2015; Romagnolo et al., 2017). In fact, genistein was shown to have positive effects against breast cancer in human breast cell lines by modifications at the molecular level (Romagnolo et al., 2017). Moreover, potential anticarcinogenic properties of genistein were also shown, as it enhanced the antiproliferative effects of other chemotherapeutic agents *in vitro* (Xu et al., 2015). Further, the beneficial properties of chrysoeriol are related to therapeutic effects in the management of type 2 diabetes mellitus, as it can act as an inhibitor of the enzymes α-amylase and lipase (Ramirez et al., 2016; Hua et al., 2018). Chrysoeriol can also exert antioxidant activity such as inhibition of lipid peroxidation and superoxidase or scavenging of DPPH radicals (Mishra et al., 2003). In total, one slice (50 g) of bread fortified with either lupin microgreens or lupin leaves contained on average 3–8 mg of flavonoids.

### Identification and quantification of carotenoids and chlorophylls in microgreens and leaves of pea and lupin

**Figure 4:**
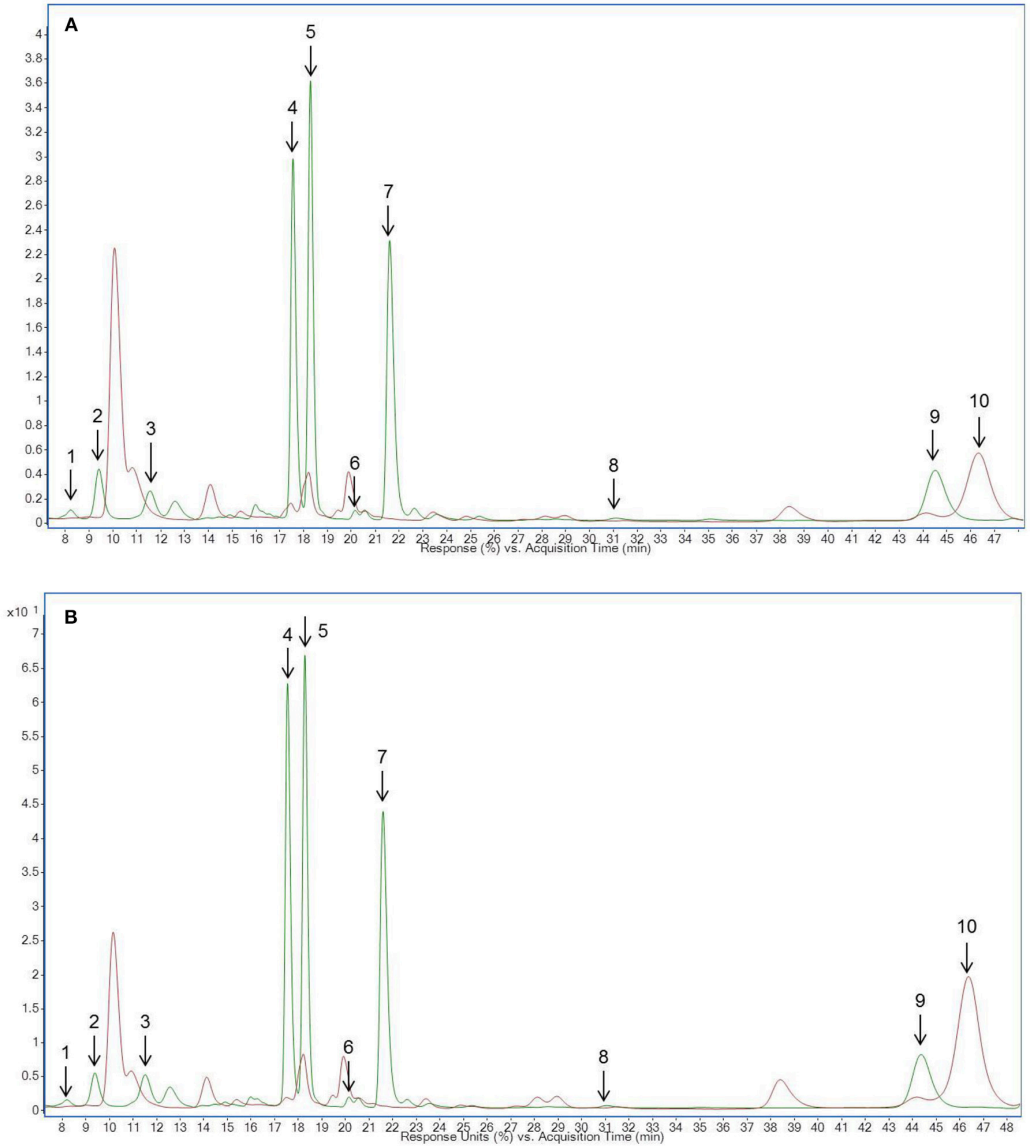
HPLC chromatogram of carotenoid and chlorophyll metabolites in **(A)** pea microgreens (green) vs. pea microgreen bread (brown) and **(B)** pea leaves (green) vs. pea leaf bread (brown). Intensity in mAU, extract at 440 nm. The bread contains 10.7% plant material, hence a dilution factor of 10.7% has to be taken into account. **(A)** Compound are numbered in chronological order 1: Neoxanthin derivative 2, 2: Violaxanthin derivative 1, 3: Neoxanthin derivative 1, 4: Chlorophyll b, 5: Lutein, 6: Zeaxanthin, 7: Chlorophyll a, 8: α-Carotene, 9: β-Carotene, 10: Pheophytin **(B)** Compound are numbered in chronological order 1: Neoxanthin derivative 2, 2: Violaxanthin derivative 1, 3: Neoxanthin derivative 1, 4: Chlorophyll b, 5: Lutein, 6: Zeaxanthin, 7: Chlorophyll a, 8: α-Carotene, 9: β-Carotene, 10: Pheophytin.

**Figure 5:**
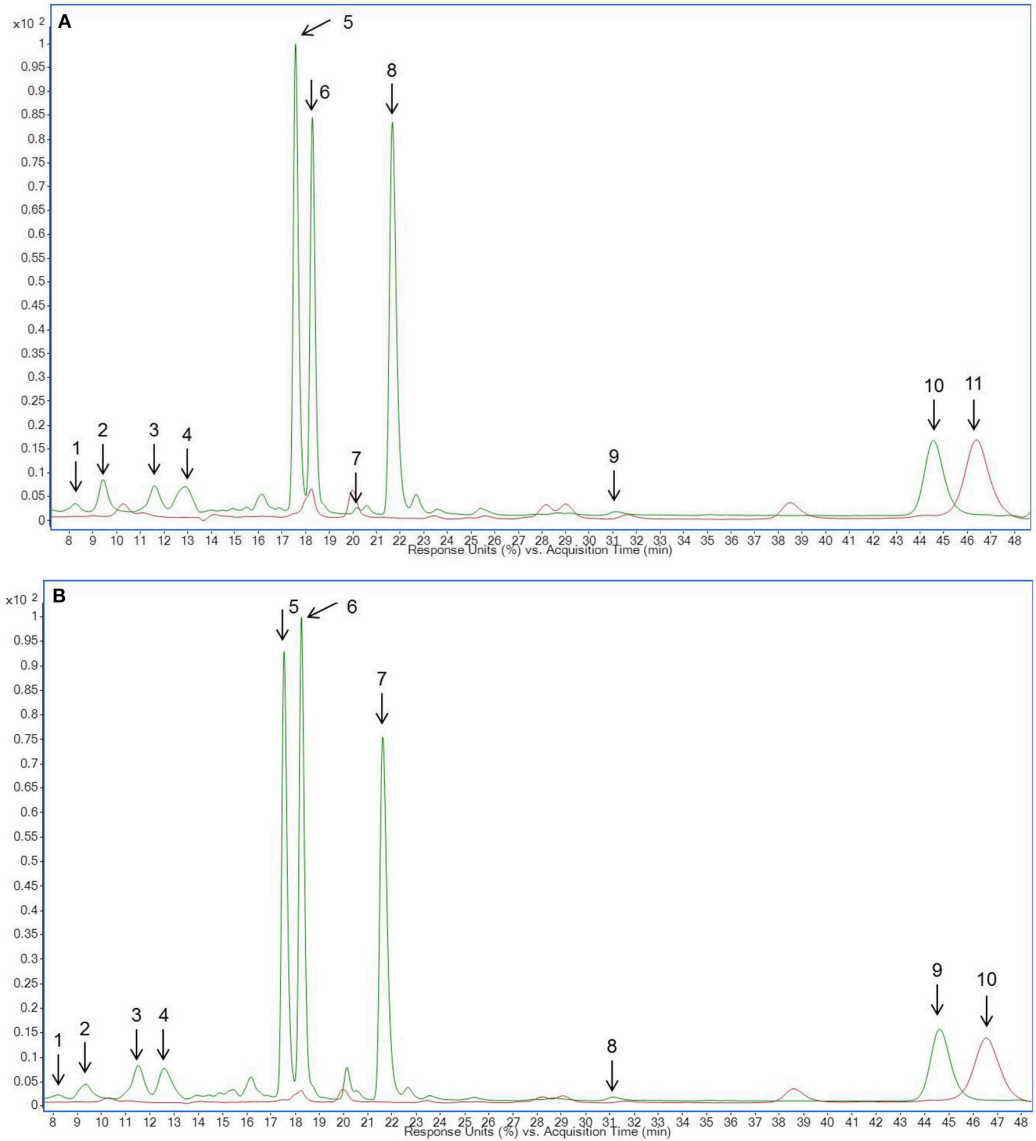
HPLC chromatogram of carotenoid and chlorophyll metabolites in **(A)** lupin microgreens (green) vs. lupin microgreen bread (brown) and **(B)** lupin leaves (green) vs. lupin leaf bread (brown). Intensity in mAU, extract at 440 nm. The bread contains 10.7% plant material, hence a dilution factor of 10.7% has to be taken into account. **(A)** 1: Neoxanthin derivative 2, 2: Violaxanthin derivative 1, 3: Neoxanthin derivative 1, 4: Violaxanthin derivative 2, 5: Chlorophyll b, 6: Lutein, 7: Zeaxanthin, 8: Chlorophyll a, 9: α-Carotene, 10: β-Carotene, 11: Pheophytin **(B)** 1: Neoxanthin derivative 2, 2: Violaxanthin derivative 1, 3: Neoxanthin derivative 1, 4: Violaxanthin derivative 2, 5: Chlorophyll b, 6: Lutein, 7: Chlorophyll a, 8: α-Carotene, 9: β-Carotene, 10: Pheophytin.

All experiments (plant growth, baking) were carried out in two replicates. The initial values of carotenoids and chlorophylls in the plants differed between these two independent experiments due to biological variations. Thus, to achieve improved clarity, values of the first and second experiment, are stated separately. Carotenoids and chlorophylls were analyzed in microgreens and leaves of pea and lupin. Both substance classes were identified by means of retention time, specific mass (*m/z*), and UV absorptions maxima obtained by UHPLC-ToF-MS (Figures 4, 5, Supplemental Table S3). Lutein, β-carotene, zeaxanthin, neoxanthin derivative1, neoxanthin derivate 2, violaxanthin, as well as chlorophyll a and chlorophyll b were identified. In general, the concentration of all carotenes and chlorophylls increased in leaves of pea and lupin compared to their corresponding microgreens (Tables 3, 4). In pea the total carotenoid concentration in the first experiment of microgreens was 135 μg g^−1^ FW and 250 μg g^−1^ FW in leaves (Table 3A). In the second experiment, the total carotenoid concentration in microgreens was 264 μg g^−1^ FW and 346 μg g^−1^ FW in pea leaves (Table 3B). In lupin, the total carotenoid concentration ranged from 133 μg g^−1^ FW in microgreens to 194 μg g^−1^ FW in leaves in the first experiment (Table 4A). In the second experiment, the total carotenoid concentration ranged from 249 μg g^−1^ FW in microgreens to 162 μg g^−1^ FW in leaves (Table 4B). The chlorophyll concentration exceeded the total carotenoid concentration in pea and lupin in both development stages. Lefsrud et al. (2007) found that β-carotene, lutein, and chlorophyll increased in kale leaves until the 2–3 week of plant development, but decreased in kale leaves older than 3 weeks. Consistent with these results, we also found that β-carotene, lutein, and chlorophyll increased in pea and lupin from microgreens to leaves. Moreover, zeaxanthin was found in lupin microgreens, but it was below the detection limit in lupin leaves, pea microgreens, and pea leaves (Tables 3, 4). In a previous study, lutein, zeaxanthin, and β-carotene were found to be the major carotenoids in lupin microgreens and leaves as well as in the seeds of four different lupin species (*L. luteus* cv. Pootalong, *L. albus* cv. Andromeda, *L. mutabilis* P26961, *L. angustifolius* cv. Kalya) (Wang et al., 2008). Furthermore, whereas lutein concentration was similar in all species, zeaxanthin, β-carotene, and indeed, the total carotenoid concentration varied among the different species (Wang et al., 2008). These findings underline the fact that the carotenoid profiles vary considerably among different lupin species and subspecies. In line with the results gained by Mroczek-Zdyrska et al. (2016) in 14-day-old lupin leaves, chlorophyll a concentration always exceeded the total carotenoid and the chlorophyll b concentration in pea and lupin microgreens and leaves (Tables 3, 4). A high chlorophyll a concentration was also described in various green vegetables such as broccoli (*Brassica oleracea* var. *silvestris*), spinach (*Spinacia oleracea*), (*Allium ampeloprasum*) (Turkmen et al., 2006). In pea, we found that carotenoid and chlorophyll concentrations were generally higher in leaves compared to microgreens. In lupin, only lutein, β-carotene, and chlorophyll concentrations were distinctly higher in lupin leaves. Comparing both microgreens and leaves of pea and lupin, carotenoid and chlorophyll concentrations were higher in both pea tissues.

**Table 3:** Average carotenoid and chlorophyll derivative concentration in Pea microgreens (7 d) and leaves (14 d): Non-processed and in the pea-enriched bread.

		**Pea microgreens**			**Pea leaves**		
		**mg g**^**−1**^ **fresh weight**					
**(A) FIRST EXPERIMENT**							
Lutein	Non-processed	71.7^A, a^	±	*7.44*	125.84^A, b^	±	*11.32*
	After baking	1.51^B^	±	*0.54*	8.66^B^	±	*0.76*
ß-Carotene	Non-processed	18.91^a^	±	*2.93*	43.15^b^	±	*3.27*
Neoxanthin derivative 1	Non-processed	13.42^a^	±	*1.34*	27.67^b^	±	*2.4*
Neoxanthin derivative 2	Non-processed	2.62^a^	±	*0.66*	5.99^b^	±	*0.8*
Violaxanthin derivative 1	Non-processed	16.2^a^	±	*1.27*	32.55^b^	±	*5.97*
Chlorophyll a	Non-processed	1017.61^a^	±	*85.71*	2019.92^b^	±	*226.82*
Chlorophyll b	Non-processed	142.6^a^	±	*17.95*	295.62^b^	±	*28.08*
Pheophytin	Non-processed		ND			ND
	After baking	137.56^A^	±	*8.73*	449.53^A^	±	*11.9*
**(B) SECOND EXPERIMENT**							
Lutein	Non-processed	130.28^A, a^	±	*18.73*	166.53^A, b^	±	*8.36*
	After baking	7.56^B^	±	*1.54*	8.48^B^	±	*0.61*
ß-Carotene	Non-processed	41.71^a^	±	*5.86*	61.88^b^	±	*3.32*
Neoxanthin derivative 1	Non-processed	25.85^a^	±	*3.9*	38.47^b^	±	*2.72*
Neoxanthin derivative 2	Non-processed	7.85^a^	±	*2.81*	6.12^a^	±	*0.57*
Violaxanthin derivative 1	Non-processed	40.35^a^	±	*8.08*	41.36^a^	±	*4.2*
Chlorophyll a	Non-processed	2018.69^a^	±	*351.13*	2227.73^a^	±	*125.2*
Chlorophyll b	Non-processed	285.69^a^	±	*44.85*	404.71^b^	±	*34.61*
Pheophytin	Non-processed		ND			ND
	After baking	263.31^A^	±	*42.11*	448.38^A^	±	*21.54*

*Results are presented as means ± SD (Italic values, n = 5) in μg g^−1^ fresh weight. ND, Not detected. Students t-test by Statistica at a significance level of p ≤ 0.05. Capital letters label significant differences between the non-processed plant material and plant material after the baking process within in the same development stage. Small letters label significant differences between non-processed microgreens and leaves*.

**Table 4:** Average carotenoid and chlorophyll derivative concentration in Lupin microgreens (7 d) and lupin leaves (14 d): Non-processed and in the lupin-enriched bread.

		**Lupin microgreens**			**Lupin leaves**		
		**mg g**^**−1**^ **fresh weight**					
**(A) FIRST EXPERIMENT**							
Lutein	Non-processed	29.64^a^	±	*5.01*	95.24^b^	±	*13.88*
ß-Carotene	Non-processed	14.8^a^	±	*1.79*	37.53^b^	±	*8.7*
Zeaxanthin	Non-processed	47.73^a^	±	*42.47*		ND
Neoxanthin derivative 1	Non-processed	19.08^a^	±	*2.47*	22.5^a^	±	*3.81*
Neoxanthin derivative 2	Non-processed	1.46^a^	±	*0.31*	2.31^b^	±	*0.55*
Violaxanthin derivative 1	Non-processed	8.23^a^	±	*1.47*	10.68^a^	±	*2.03*
Violaxanthin derivative 2	Non-processed	11.9^a^	±	*4.14*	26^b^	±	*4.43*
Chlorophyll a	Non-processed	594.03^a^	±	*63.63*	1338.33^b^	±	*220.66*
Chlorophyll b	Non-processed	87.41^a^	±	*11.09*	225.01^b^	±	*21.98*
Pheophytin	Non-processed		ND			ND
	After baking	173.1^A^	±	*45.32*	654.37^A^	±	*51.7*
**(B) SECOND EXPERIMENT**							
Lutein	Non-processed	27.02^a^	±	*18.71*	104.61^b^	±	*21.89*
ß-Carotene	Non-processed	17.78^a^	±	*6.01*	55^b^	±	*9.52*
Zeaxanthin	Non-processed	147.44^a^	±	*59.74*		ND
Neoxanthin derivative 1	Non-processed	24.01^a^	±	*2.22*	0.72^b^	±	*0.17*
Neoxanthin derivative 2	Non-processed	2.44^a^	±	*0.38*	0.45^b^	±	*0.05*
Violaxanthin derivative 1	Non-processed	10.91^a^	±	*1.18*	0.75^b^	±	*0.08*
Violaxanthin derivative 2	Non-processed	22.26^a^	±	*3.62*		ND
Chlorophyll a	Non-processed	609.1^a^	±	*178.7*	1776.03^b^	±	*332.83*
Chlorophyll b	Non-processed	74.64^a^	±	*36.78*	270.78^b^	±	*56*
Pheophytin	Non-processed		ND			ND
	After baking	204.19^A^	±	*79.02*	611.97^A^	±	*13.85*

*Results are presented as means ± SD (Italic values, n = 5) in μg g^−1^ fresh weight. ND, Not detected. Students t-test by Statistica at a significance level of p ≤ 0.05. Capital letters label significant differences between the non-processed plant material and plant material after the baking process within in the same development stage. Small letters label significant differences between non-processed microgreens and leaves*.

### Identification and quantification of carotenoids and chlorophyll metabolites in legume-enriched bread

Non-processed pea and lupin tissue from the days of breadmaking, breads fortified with pea and lupin tissue, and control breads were analyzed regarding their carotenoid and chlorophyll metabolite concentration. Neither carotenoid nor chlorophyll metabolites were detected in the control bread. With the exception of lutein, no carotenoids were found in the legume-enriched breads (Table 3, Figures 4, 5). Less than 6% of the initial lutein concentration could be recovered in the “pea microgreen bread” and the “pea leaf bread,” no lutein was found in the lupin breads (Table 3). Chlorophyll was also not detectable in pea- or in lupin-enriched bread. Earlier studies described major decreases of carotenoid respective chlorophyll concentrations in a variety of vegetables, e.g., broccoli, spinach, and green beans, due to heat treatment (Murcia et al., 2000; Turkmen et al., 2006). Pheophytin formation, simultaneously to a decrease in chlorophyll concentration, was also described earlier when boiling vegetables (e.g., green beans, spinach, and peas) (Turkmen et al., 2006). Moreover, the decrease of carotenoid and chlorophyll (respective pheophytin formation) concentrations varies between different species and increases with higher temperatures (Turkmen et al., 2006; Guillén et al., 2017). In broccoli inflorescence (*Brassica oleracea* var. Avenger) the lutein and chlorophyll concentrations were even higher in boiled vegetables compared to fresh ones (dos Reis et al., 2015). Therefore, the impact of heat treatment can be considered as species and organ dependent (dos Reis et al., 2015). In spinach leaves, broccoli, and Brussels sprouts, chlorophyll thermostability was also shown to be dependent on the specific heat treatment (Teng and Chen, 1999; Paciulli et al., 2017). In addition, aqueous heat treatments (steaming, blanching, and microwaving) led to a stronger decrease of chlorophyll b concentrations compared to chlorophyll a ones, whereas dry heat treatments (baking) had a similar effect on both chlorophyll a and b levels (Guillén et al., 2017; Paciulli et al., 2017). Accordingly, chlorophyll a and b concentrations were completely depleted in both pea- and lupin-enriched bread. Carotenoid and chlorophyll concentrations were shown to decrease with longer heat treatments, thereby pheophytin formation increases (Teng and Chen, 1999). In accordance with previous investigations, we also found increased pheophytin concentrations in pea- and lupin-enriched bread as a result of chlorophyll depletion (Tables 3, 4, Figures 4, 5) (Turkmen et al., 2006). No pheophytin was detected in non-processed pea and lupin tissue. In the “pea microgreen bread” from the first experiment, we found 137 μg g^−1^ FW of pheophytin and 450 μg g^−1^ FW in the “pea leaf bread” (Table 3A). In “pea microgreen bread” from the second experiment, we found 263 μg g^−1^ FW of pheophytin and 448 μg g^−1^ FW in “pea leaf bread” (Table 3B). In “lupin microgreen and leaf bread” from the first experiment, we found 173 and 654 μg g^−1^ FW of pheophytin, respectively (Table 4A). In the “lupin microgreen and leaf breads” from the second experiment, 204 and 612 μg g^−1^ FW of pheophytin were found, respectively (Table 4B). Even though the breadmaking process led to an almost complete loss of carotenoids and chlorophylls, some health-promoting properties were maintained due to increased pheophytin formation, as pheophytin is thought to have similar health-promoting effects as chlorophyll, e.g., anti-inflammatory and antioxidant activities (Hsu et al., 2013; Park et al., 2014). Thus, bread enriched with leaves, either from pea or lupin, had a higher pheophytin concentration compared to the breads with the corresponding microgreens.

## Conclusion

Pea and lupin could provide the consumer with health-promoting secondary plant metabolites such as carotenoids, chlorophylls, and flavonoids. The present study showed that the developmental stage has a distinct impact on the secondary plant metabolite profile and has to be taken into account prior to use plant material for further food processes. For example, the total concentration of carotenoids, chlorophylls, and flavonoids was generally higher in pea and lupin leaves compared to their corresponding microgreens. Hence, the use of pea and lupin leaves for enriching food products should be favored in order to enhance health-promoting effects. Moreover, quercetin glycosides were predominant in both investigated pea tissues, suggesting that pea microgreens as well as leaves are good sources of quercetin. Although the total flavonoid concentration was higher in lupin leaves compared to microgreens, the genistein concentration was higher in lupin microgreens. However, lupin leaves accumulated higher amounts of chrysoeriol. Hence, recommendations for lupin consumption could be tailored to the specific needs of the consumer such as for anticarcinogenic properties (lupin microgreens rich in genistein) or antidiabetic benefits (lupin leaves rich in chrysoeriol). In most cases, vegetables are consumed after thermal processing (cooked, steamed, or fried) which is thought to lower the nutritional value of such cooked vegetables. In the present study, we found that baking of fresh plant material in bread led to relatively low losses of flavonoids, major depletion of carotenoid and chlorophyll concentrations, but also an increase in pheophytin formation. Nevertheless, breads enriched with plant material from pea or lupin could result in increased consumption of desired secondary plant metabolites. In detail, pea-enriched breads could provide the consumer with increased concentration of health-promoting kaempferol and quercetin derivatives. In addition, genistein-containing “lupin microgreen bread” could have strong health benefits, particularly for women. Further, “lupin leaf bread” rich in chrysoeriol could be used as a substitute for traditional bread to consumers with a type 2 diabetes mellitus. Collectively, this study has shown that dependent on the chosen plant material, it is possible to produce bread enriched with health-promoting secondary plant metabolites tailored for specific consumer needs.

## Author contributions

MS, SN, AV, SR, and RK contributed conception and design of the study; RK, SB, SN organized the database; RK performed the statistical analysis; RK wrote the first draft of the manuscript; SB, SR, MS, SN wrote sections of the manuscript. All authors contributed to manuscript revision, read, and approved the submitted version.

### Conflict of interest statement

The authors declare that the research was conducted in the absence of any commercial or financial relationships that could be construed as a potential conflict of interest.

## References

[B1] Acker L. Bergner K. G. Diemair W. Heimann W. Kiermeier F. Schormüller J. (2013). Brot, Backwaren und Hilfsmittel. Berlin; Heidelberg: Springer-Verlag.

[B2] Aisyah S. Vincken J.-P. Andini S. Mardiah Z. Gruppen H. (2016). Compositional changes in (iso) flavonoids and estrogenic activity of three edible lupinus species by germination and rhizopus-elicitation. Phytochem 122, 65–75. 10.1016/j.phytochem.2015.12.015 26749476

[B3] Andersen O. M. Markham K. R. (2006). Flavonoids: Chemistry, Biochemistry and Applications. Boca Raton, FL: CRC Press.

[B4] Baardseth P. Bjerke F. Martinsen B. K. Skrede G. (2010). Vitamin, C., total phenolics and antioxidative activity in tip-cut green beans (*Phaseolus vulgaris*) and swede rods (*Brassica napus* var. napobrassica) processed by methods used in catering. J. Sci. Food Agric. 90, 1245–1255. 10.1002/jsfa.3967 20394008

[B5] Baldermann S. Yang Z. Sakai M. Fleischmann P. Morita A. Todoroki Y.. (2013). Influence of exogenously applied abscisic acid on carotenoid content and water uptake in flowers of the tea plant (*Camellia sinensis*). J. Sci. Food Agric. 93, 1660–1664. 10.1002/jsfa.5944 23152164

[B6] Buchner N. Krumbein A. Rohn S. Kroh L. W. (2006). Effect of thermal processing on the flavonols rutin and quercetin. Rapid Commun. Mass Spectrom. 20, 3229–3235. 10.1002/rcm.2720 17016866

[B7] Bussler S. Herppich W. B. Neugart S. Schreiner M. Ehlbeck J. Rohn S. (2015). Impact of cold atmospheric pressure plasma on physiology and flavonol glycoside profile of peas (*Pisum sativum* ‘Salamanca’). Food Res. Int. 76, 132–141. 10.1016/j.foodres.2015.03.045

[B8] Chaaban H. Ioannou I. Chebil L. Slimane M. Gérardin C. Paris C. (2017). Effect of heat processing on thermal stability and antioxidant activity of six flavonoids. J. Food Process. Preserv. 41:e13203 10.1111/jfpp.13203

[B9] dos Reis L. C. R. De Oliveira V. R. Hagen M. E. K. Jablonski A. Flôres S. H. De Oliveira Rios A. (2015). Carotenoids, flavonoids, chlorophylls, phenolic compounds and antioxidant activity in fresh and cooked broccoli (*Brassica oleracea* var. Avenger) and cauliflower (*Brassica oleracea* var. Alphina F1). LWT - Food Sci. Techno. 63, 177–183. 10.1016/j.lwt.2015.03.089

[B10] Duenas M. Hernandez T. Estrella I. Fernandez D. (2009). Germination as a process to increase the polyphenol content and antioxidant activity of lupin seeds (*Lupinus angustifolius* L.). Food Chem. 117, 599–607. 10.1016/j.foodchem.2009.04.051

[B11] Ewald C. Fjelkner-Modig S. Johansson K. Sjöholm I. Åkesson B. (1999). Effect of processing on major flavonoids in processed onions, green beans, and peas. Food Chem. 64, 231–235. 10.1016/S0308-8146(98)00136-8

[B12] Fiol M. Weckmüller A. Neugart S. Schreiner M. Rohn S. Krumbein A.. (2013). Thermal-induced changes of kale's antioxidant activity analyzed by HPLC–UV/Vis-online-TEAC detection. Food Chem. 138, 857–865. 10.1016/j.foodchem.2012.10.101 23411188

[B13] Frede K. Ebert F. Kipp A. P. Schwerdtle T. Baldermann S. (2017). Lutein activates the transcription factor Nrf2 in human retinal pigment epithelial cells. J. Agric. Food Chem. 65, 5944–5952. 10.1021/acs.jafc.7b01929 28665123

[B14] García-López P. Kachlicki P. Zamora-Natera F. Ruiz-Moreno J. Stobiecki M. (2006). Profiling isoflavone conjugates in different organs of *Lupinus exaltatus Zucc*. Phytochem. Anal 17, 184–191. 10.1002/pca.904 16749426

[B15] Grün I. U. Adhikari K. Li C. Li Y. Lin B. Zhang J.. (2001). Changes in the profile of genistein, daidzein, and their conjugates during thermal processing of tofu. J. Agric. Food Chem. 49, 2839–2843. 10.1021/jf010028+ 11409975

[B16] Guillén S. Mir-Bel J. Oria R. Salvador M. L. (2017). Influence of cooking conditions on organoleptic and health-related properties of artichokes, green beans, broccoli and carrots. Food Chem. 217, 209–216. 10.1016/j.foodchem.2016.08.067 27664628

[B17] Hostetler G. L. Riedl K. M. Schwartz S. J. (2013). Effects of food formulation and thermal processing on flavones in celery and chamomile. Food Chem. 141, 1406–1411. 10.1016/j.foodchem.2013.04.051 23790931 PMC3850025

[B18] Hsu C.-Y. Chao P.-Y. Hu S.-P. Yang C.-M. (2013). The antioxidant and free radical scavenging activities of chlorophylls and pheophytins. FNS 4, 1–8. 10.4236/fns.2013.48A001

[B19] Hua F. Zhou P. Wu H.-Y. Chu G.-X. Xie Z.-W. Bao G.-H. (2018). Inhibition of α-glucosidase and α-amylase by flavonoid glycosides from Lu'an GuaPian tea: molecular docking and interaction mechanism. Food Funct. [Epub ahead of print]. 10.1039/c8fo00562a. 29989631

[B20] Juániz I. Ludwig I. A. Huarte E. Pereira-Caro G. Moreno-Rojas J. M. Cid C.. (2016). Influence of heat treatment on antioxidant capacity and (poly)phenolic compounds of selected vegetables. Food Chem. 197, 466–473. 10.1016/j.foodchem.2015.10.139 26616976

[B21] Kachlicki P. Einhorn J. Muth D. Kerhoas L. Stobiecki M. (2008). Evaluation of glycosylation and malonylation patterns in flavonoid glycosides during LC/MS/MS metabolite profiling. J. Mass Spectrom. 43, 572–586. 10.1002/jms.1344 18074333

[B22] Kashyap D. Sharma A. Tuli H. S. Sak K. Punia S. Mukherjee T. K. (2017). Kaempferol–A dietary anticancer molecule with multiple mechanisms of action: recent trends and advancements. J. Funct. Foods 30, 203–219. 10.1016/j.jff.2017.01.022 PMC7104980 32288791

[B23] Katagiri Y. Ibrahim R. K. Tahara S. (2000). HPLC analysis of white lupin isoflavonoids. Biosci. Biotechnol. Biochem. 64, 1118–1125. 10.1271/bbb.64.1118 10923779

[B24] Krems C. Walter C. Heuer T. Hoffmann I. (2013). Nationale Verzehrsstudie II Lebensmittelverzehr und Nährstoffzufuhr auf Basis von 24h-Recalls. Max Rubner-Institut.

[B25] Lefsrud M. Kopsell D. Wenzel A. Sheehan J. (2007). Changes in kale (*Brassica oleracea* L. var. acephala) carotenoid and chlorophyll pigment concentrations during leaf ontogeny. Sci. Hortic. 112, 136–141. 10.1016/j.scienta.2006.12.026

[B26] Lin J. Zhou W. (2018). Role of quercetin in the physicochemical properties, antioxidant and antiglycation activities of bread. J. Funct. Foods 40, 299–306. 10.1016/j.jff.2017.11.018

[B27] Mishra B. Priyadarsini K. I. Kumar M. S. Unnikrishnan M. Mohan H. (2003). Effect of O-glycosilation on the antioxidant activity and free radical reactions of a plant flavonoid, chrysoeriol. Bioorg. Med. Chem. 11, 2677–2685. 10.1016/S0968-0896(03)00232-3 12788341

[B28] Mroczek-Zdyrska M. Kornarzynski K. Pietruszewski S. Gagos M. (2016). Stimulation with a 130-mT magnetic field improves growth and biochemical parameters in lupin (*Lupinus angustifolius* L.). Turk. J. Biol. 40, 699–705. 10.3906/biy-1504-19

[B29] Murcia M. A. Lápez-Ayerra B. Martínez-Tomé M. García-Carmona F. (2000). Effect of industrial processing on chlorophyll content of broccoli. J. Sci. Food Agric 80, 1447–1451. 10.1002/1097-0010(200008)80:10<1447::AID-JSFA670>3.0.CO;2-X

[B30] Nascimento P. L. Nascimento T. C. Ramos N. S. Silva G. R. Gomes J. E. G. Falcão R. E.. (2014). Quantification, antioxidant and antimicrobial activity of phenolics isolated from different extracts of *Capsicum frutescens* (*Pimenta Malagueta*). Molecules 19, 5434–5447. 10.3390/molecules19045434 24879587 PMC6271728

[B31] Neugart S. Rohn S. Schreiner M. (2015). Identification of complex, naturally occurring flavonoid glycosides in *Vicia faba* and *Pisum sativum* leaves by HPLC-DAD-ESI-MS n and the genotypic effect on their flavonoid profile. Food Res. Int. 76, 114–121. 10.1016/j.foodres.2015.02.021

[B32] Paciulli M. Palermo M. Chiavaro E. Pellegrini N. (2017). Chlorophylls and colour changes in cooked vegetables, in Fruit and Vegetable Phytochemicals: Chemistry and Human Health, 2nd Edn., ed Yahia E. M. (Chichester, UK; Hoboken, NJ: John Wiley & Sons, Ltd), 703–719. 10.1002/9781119158042.ch31

[B33] Park S. Choi J. J. Park B.-K. Yoon S. J. Choi J. E. Jin M. (2014). Pheophytin a and chlorophyll a suppress neuroinflammatory responses in lipopolysaccharide and interferon-γ-stimulated BV2 microglia. Life Sci. 103, 59–67. 10.1016/j.lfs.2014.04.003 24735958

[B34] Pot G. K. Prynne C. J. Almoosawi S. Kuh D. Stephen A. M. (2015). Trends in food consumption over 30 years: evidence from a British birth cohort. Eur. J. Clin. Nutr. 69, 817–823. 10.1038/ejcn.2014.223 25351642 PMC4926956

[B35] Ramirez G. Zamilpa A. Zavala M. Perez J. Morales D. Tortoriello J. (2016). Chrysoeriol and other polyphenols from *Tecoma stans* with lipase inhibitory activity. J. Ethnopharmacol. 185, 1–8. 10.1016/j.jep.2016.03.014 26970570

[B36] Ranawana V. Campbell F. Bestwick C. Nicol P. Milne L. Duthie G. (2016). Breads fortified with freeze-dried vegetables: quality and nutritional attributes. part ii: breads not containing oil as an ingredient. Foods 5:62 10.3390/foods5030062 PMC5224574 28231114

[B37] Rohn S. Buchner N. Driemel G. Rauser M. Kroh L. W. (2007). Thermal degradation of onion quercetin glucosides under roasting conditions. J. Agric. Food Chem. 55, 1568–1573. 10.1021/jf063221i 17263552

[B38] Romagnolo D. F. Donovan M. G. Papoutsis A. J. Doetschman T. C. Selmin O. I. (2017). Genistein prevents BRCA1 CpG methylation and proliferation in human breast cancer cells with activated aromatic hydrocarbon receptor. Curr. Dev. Nutr. 1:e000562. 10.3945/cdn.117.000562 29955703 PMC5998349

[B39] Saito K. Yonekura-Sakakibara K. Nakabayashi R. Higashi Y. Yamazaki M. Tohge T.. (2013). The flavonoid biosynthetic pathway in Arabidopsis: structural and genetic diversity. Plant Physiol. Biochem. 72, 21–34. 10.1016/j.plaphy.2013.02.001 23473981

[B40] Santos J. Herrero M. Mendiola J. A. Oliva-Teles M. T. Ibanez E. Delerue-Matos C. (2014). Assessment of nutritional and metabolic profiles of pea shoots: the new ready-to-eat baby-leaf vegetable. Food Res. Int. 58, 105–111. 10.1016/j.foodres.2014.01.062

[B41] Schröter D. Baldermann S. Schreiner M. Witzel K. Maul R. Rohn S.. (2017). Natural diversity of hydroxycinnamic acid derivatives, flavonoid glycosides, carotenoids and chlorophylls in leaves of six different amaranth species. Food Chem. 267, 376–386. 10.1016/j.foodchem.2017.11.043 29934181

[B42] Šibul F. Orčić D. Vasić M. Anačkov G. Nadpal J. Savić A. Mimica-Dukić N. (2016). Phenolic profile, antioxidant and anti-inflammatory potential of herb and root extracts of seven selected legumes. Ind. Crops Prod. 83, 641–653. 10.1016/j.indcrop.2015.12.057

[B43] Teng S. Chen B. (1999). Formation of pyrochlorophylls and their derivatives in spinach leaves during heating. Food Chem. 65, 367–373. 10.1016/S0308-8146(98)00237-4

[B44] Turkmen N. Poyrazoglu E. S. Sari F. Sedat Velioglu Y. (2006). Effects of cooking methods on chlorophylls, pheophytins and colour of selected green vegetables. Int. J. Food Sci. Technol. 41, 281–288. 10.1111/j.1365-2621.2005.01061.x

[B45] Ungar Y. Osundahunsi O. F. Shimoni E. (2003). Thermal stability of genistein and daidzein and its effect on their antioxidant activity. J. Agric. Food Chem. 51, 4394–4399. 10.1021/jf034021z 12848516

[B46] Wang S. Errington S. Yap H. H. (2008). Studies on carotenoids from lupin seeds, in Lupins for Health and Wealth'Proceedings of the 12th International Lupin Conference (Fremantle), 14–18.

[B47] Wang W. Sun C. Mao L. Ma P. Liu F. Yang J. (2016). The biological activities, chemical stability, metabolism and delivery systems of quercetin: a review. Trends Food Sci. Technol. 56, 21–38. 10.1016/j.tifs.2016.07.004

[B48] Wojakowska A. Piasecka A. Garcia-Lopez P. M. Zamora-Natera F. Krajewski P. Marczak L.. (2013). Structural analysis and profiling of phenolic secondary metabolites of Mexican lupine species using LC-MS techniques. Phytochemistry 92, 71–86. 10.1016/j.phytochem.2013.04.006 23642387

[B49] World Health Organisation (2018). A Healthy Lifestyle. Available online at: http://www.euro.who.int/en/health-topics/disease-prevention/nutrition/a-healthy-lifestyle (Accessed January 8, 2018).

[B50] Xu M.-L. Liu J. Zhu C. Gao Y. Zhao S. Liu W. (2015). Interactions between soy isoflavones and other bioactive compounds: a review of their potentially beneficial health effects. Phytochem. Rev. 14, 459–467. 10.1007/s11101-015-9398-0

[B51] Xu Z. Wu Q. Godber J. S. (2002). Stabilities of daidzin, glycitin, genistin, and generation of derivatives during heating. J. Agric. Food Chem. 50, 7402–7406. 10.1021/jf025626i 12452666

[B52] Zhang L.-N. Cao P. Tan S.-H. Gu W. Shi L. Zhu H.-L. (2008). Synthesis and antimicrobial activities of 7-O-modified genistein derivatives. Eur. J. Med. Chem. 43, 1543–1551. 10.1016/j.ejmech.2007.09.008 17977623

[B53] Zhao J. Dixon R. A. (2010). The ‘ins’ and ‘outs’ of flavonoid transport. Trends Plant Sci. 15, 72–80. 10.1016/j.tplants.2009.11.006 20006535

